# Phylogenomics resolves long-standing questions about the affinities of an endangered Corsican endemic fly

**DOI:** 10.1093/jisesa/ieae073

**Published:** 2024-07-25

**Authors:** Pierfilippo Cerretti, Liping Yan, Sujatha Narayanan Kutty, Krzysztof Szpila, Dario Nania, Roxana Tintea, Maurizio Mei, Thomas Pape

**Affiliations:** Department of Biology and Biotechnologies “Charles Darwin”, Sapienza University of Rome, Roma, Italy; School of Ecology and Nature Conservation, Beijing Forestry University, Beijing, China; Tropical Marine Science Institute, National University of Singapore, Singapore, Singapore; Faculty of Biological and Veterinary Sciences, Department of Ecology and Biogeography, Nicolaus Copernicus University in Toruń, Toruń, Poland; Department of Biology and Biotechnologies “Charles Darwin”, Sapienza University of Rome, Roma, Italy; Department of Biology and Biotechnologies “Charles Darwin”, Sapienza University of Rome, Roma, Italy; Department of Biology and Biotechnologies “Charles Darwin”, Sapienza University of Rome, Roma, Italy; Natural History Museum of Denmark, University of Copenhagen, Copenhagen, Denmark

**Keywords:** Calliphoridae, Diptera, IUCN threatened category, *Nesodexia*, Rhinophorinae

## Abstract

Recent studies on oestroidean Diptera (Brachycera) are providing a comprehensive and nuanced understanding of the evolutionary history of this remarkably diverse clade of holometabolous insects. The Oestroidea, which includes formidable pests such as various blowflies, botflies, and flesh flies that infest livestock, pets and humans, are mostly composed of beneficial species that act as scavengers or parasitoids on various pest insects. In our research, we used genomic methods to elucidate the phylogenetic position of *Nesodexia corsicana* Villeneuve, 1911 (Diptera: Calliphoridae), a mysterious oestroid species endemic to Corsica and characterized by distinctive morphological features that have puzzled taxonomists for years. Contrary to initial hypotheses, our results place *Nesodexia* Villeneuve, 1911 within the Calliphoridae subfamily Rhinophorinae, a small lineage of terrestrial isopod parasitoids. Through detailed morphological analysis of adults of both sexes and eggs, we uncovered significant insights consistent with our phylogenomic reconstruction. The unique morphological features of the species, coupled with its restricted and fragmented habitat, highlight its potential conservation importance. We delineated the area of occupancy for *N. corsicana* and assessed its “threatened” category using specific IUCN Red List criteria. In addition, we mapped the available habitat within its range and determined potential key biodiversity areas (KBA) triggered by *N. corsicana*. New potential KBAs are only partially covered by the Corsican Regional Park. Finally, we mapped the distribution of habitats on the island to assess the potential distribution of the species beyond its currently known geographic range.

## Graphical Abstract

**Graphical Abstract:**
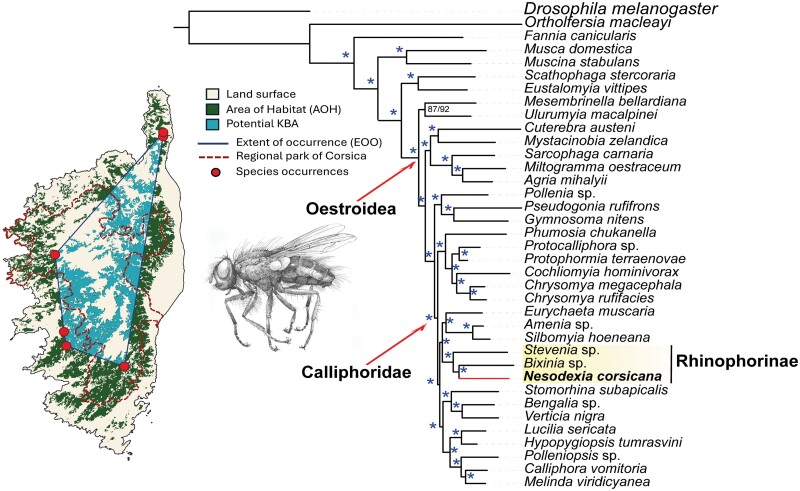

## Introduction

Ecosystems of large islands are often characterized by a rich assemblage of endemic species, which show unique morphological and functional traits, and they are often dependent on particular and localized environmental resources that determine their spatial distribution and population dynamics (Gillespie and Roderick 2002). Increasing human pressure on islands, with the associated changes in environmental conditions, pose a serious threat to these vulnerable biotic elements. In fact, islands are unlikely to provide refugia during environmental transformations, and island biotas are in general more susceptible to having their genetic diversity eroded when habitat shrinkage and fragmentation reduce the range and size of populations (Russell and Kueffer 2019). The risk of losing unique components of biodiversity before we even understand their ecological roles and evolution is now considered not only an ethical and cultural issue, but also an economic one (Russell and Kueffer 2019, World Economic Forum Global Risk Report 2024). The study of endemic island plants and animals has been key to understanding evolution and its mechanisms (Kaneshiro 1983, Brown et al. 2013, Ketmaier and Caccone 2013), and the loss of unique components of these complex systems can have serious consequences for the quality of human life.

Endemic island species often have distinctive morphological and functional characteristics (Rozzi et al. 2023). For example, island dwarfism and gigantism have been described and documented in many vertebrates (including humans) and some insect groups (Chown and Gaston 2010, Poulakakis et al. 2012, Sota et al. 2020, Benítez-López et al. 2021), but island endemics, regardless of size, can also be so morphologically divergent from their phylogenetically closest relatives that their phylogenetic placement is difficult to trace (Shaphiro et al. 2002). The phylogenetic signal can be blurred when endemic organisms are exceptionally autapomorphic, but also when they are phylogenetic relicts retaining several plesiomorphic features, which have been modified in their close relatives (Grandcolas et al. 2014).

Located in the northwest of the Mediterranean, Corsica is the fourth largest island in the Mediterranean. It is considered a fragmented island as the Corsica-Sardinia block detached from mainland Europe by drifting along a counterclockwise rotational trajectory during the Miocene (Speranza et al. 2002). Corsica is one of Europe’s biodiversity hotspots, supporting a diverse flora and fauna rich in endemic elements (Médail and Quézel 1999, Myers et al. 2000, Pape et al. 2015, Touroult et al. 2023). As a result, Corsica is characterized by a wealth of unique habitats, several of which are priority sites listed in Annex I of the European Habitats Directive (https://environment.ec.europa.eu/topics/nature-and-biodiversity/habitats-directive_en), and high proportion of endemism (Dapporto 2009, Dapporto and Dennis 2009, Ketmaier and Caccone 2013).

This study deals with the phylogeny, systematics, and conservation status of a neglected component of the island’s unique biota, the endemic blow fly species *Nesodexia corsicana*Villeneuve, 1911. *N. corsicana* belongs to the Oestroidea, a large clade of calyptrates that includes parasites of vertebrates (e.g., all Oestridae and some Calliphorinae, Chrysomyinae, Sarcophaginae, Paramacronychiinae), parasitoids or predators of annelids, arthropods, and mollusks (e.g., all Rhinophorinae, Polleniidae, Tachinidae and some Ameniinae, Calliphorinae, Sarcophaginae) and scavengers (many Calliphoridae and Sarcophagidae) (Pape et al. 2009, Marshall 2012). Villeneuve (1911) erected the genus *Nesodexia* for the single species *N. corsicana*, classifying it as a dexiine tachinid but suggesting a possible sarcophagid affinity. Villeneuve (1911) did not provide much information on the habitat or behavior of *Nesodexia*, other than briefly noting that this species was abundant on flowering *Sambucus ebulus* Linnaeus. Since the large collecting expedition, which produced the original specimens, only a few other specimens have been collected and preserved in museum collections, contributing to an aura of mystery felt by many oestroid workers about this taxon. Villeneuve (1920) later classified *N. corsicana* in the Calliphoridae, which has been followed by Séguy (1928), Zumpt (1956), and Rognes (1991, 1997, 1998). The latter author discussed its phylogenetic affinities using cladistic arguments and concluded that it should possibly be placed in the Polleniinae (then a subfamily of Calliphoridae) pending further study. More recently, Cerretti et al. (2019) elevated Polleniidae to full family rank, and Gisondi et al. (2020) followed Rognes (1991) in treating *Nesodexia* as a Polleniidae.

Our phylogenomic analyses (see below) have now revealed that *Nesodexia* is a woodlouse fly (Diptera, Calliphoridae, Rhinophorinae)—a small clade of oniscoidean isopod parasitoids—overturning previous hypotheses on its phylogenetic affinities and suggesting that it probably develops as an endoparasitoid of sowbugs. This finding prompted a detailed morphological study of adults of both sexes, leading to the present detailed redescription of the taxon and a complete reinterpretation of its features in light of our phylogenomic results.

*Nesodexia corsicana* shows a mix of features that make it interesting for several reasons: its morphology has been indecipherable, if not misleading, to the extent that dipterists have long considered it a phylogenetic enigma; it is among the few blow flies with asymmetric copulatory organ; and its size indicates a host-association with one of the large, endemic woodlice. Furthermore, no other blow fly has (as yet) attracted the attention of conservation biologists, but *N. corsicana* is part of the unique, high-endemicity Corsican biota (Fois et al. 2022) and is apparently restricted to the increasingly threatened Mediterranean sclerophyll (Bussotti et al. 2014). Despite several previous expeditions to Corsica and the nearby islands of Sardinia (e.g., Cerretti et al. 2009, Rognes 2011), as well as the Tuscan archipelago (P.C., unpublished), no additional specimen records for *Nesodexia* have ever been obtained. These characteristics highlight the potential conservation importance of *N. corsicana* and prompted us to investigate further by delineating the area of occupancy (AOO) for this species and assessing its conservation status using specific IUCN Red List criteria. In addition, we mapped the distribution of suitable habitats on the island to predict the potential distribution of the species beyond its currently known geographic range. We also identified potential key biodiversity areas (KBAs) indicated by the presence of *N. corsicana* and found that potential KBAs are only partially covered by the Corsican Regional Park.

## Materials and Methods

### DNA Extraction

DNA was extracted from a specimen of *N. corsicana* collected during a field expedition in early July 2021 and immediately preserved in pure ethanol (see “Material examined” below). Extraction was performed using the Qiagen DNeasy Tissue kit (Qiagen) according to the manufacturer’s instructions, and the library was prepared using the VAHTS Universal DNA Library Prep Kit for Illumina ND607 (Vazyme biotech, Nanjing, China). Fifteen Gb of 150PE data output was targeted and sequenced on an Illumina NovaSeq6000 sequencing platform (NCBI run accession: SRR20824209).

### Assemblies and Orthology Prediction

De novo assembly of genome data was carried out in CLC Genomics Workbench 7.5.1 (https://digitalinsights.qiagen.com/products-overview/discovery-insights-portfolio/analysis-and-visualization/qiagen-clc-genomics-workbench/) [initially trimmed (limit: 0.001); *denovo* option parameters (word size = 50, bubble size = 50–150, identity fraction = 1, length fraction = 1)]. All assembled contigs were checked for contaminant sequences through a VecScreen contamination screen by the National Center for Biotechnology Information. Contigs with > 10× coverage were used for all downstream analyses. Orthology prediction, postprocessing, and dataset preparation followed the methods outlined in Kutty et al. (2019).

Orthology prediction was carried out using Orthograph 0.7.1 (Petersen et al. 2017) based on an orthologous reference set with 3,288 single-copy protein-encoding genes comprising clusters of orthologous sequences of the following five reference species for which official gene sets are available, *Anopheles gambiae* Giles, *Bombyx mori* (Linnaeus), *Drosophila melanogaster* Meigen, *Mayetiola destructor* (Say), and *Tribolium castaneum* Herbst (see Kutty et al. 2019). The dataset was assembled following similar steps from Kutty et al. (2019) utilizing custom *perl* scripts used in (Misof et al. 2014). Additional information is given in Supplementary File S1. The final single-copy protein-encoding dataset and a corresponding optimized nucleotide dataset were generated with custom-made perl-scripts.

### Phylogenetic Reconstruction

The analyses of currently available calliphorid phylogenomic data (Table 1) were performed following Kutty et al. (2019) and Yan et al. (2021). Maximum likelihood (ML) trees were inferred using IQTREE version 2.0.5 (Minh et al. 2020) based on amino acid and 1st and 2nd-codon position (NT12) matrices. The self-implemented ModelFinder was used to estimate the best model for each gene (Kalyaanamoorthy et al. 2017) following the Akaike Information Corrected Criterion score (Hurvich and Tsai 1989). Branch support was evaluated with ultrafast bootstrap resampling analysis.

**Table 1.** Taxon sampling for phylogenomic analyses in the present study

Superfamily	Family	Subfamily	Species	Accession/version and reference
	Drosophilidae		*Drosophila melanogaster*	FBgn0015805
Hippoboscoidea	Hippoboscidae		*Ortholfersia macleayi*	SRR1695374
Muscoid grade	Anthomyiidae		*Eustalomyia vittipes*	SRR1695308
	Fanniidae		*Fannia canicularis*	SRR8236841
	Muscidae		*Muscina stabulans*	SRR6724172
			*Musca domestica*	GCA_000371365
	Scathophagidae		*Scathophaga stercoraria*	SRR8236843
Oestroidea	Calliphoridae	Ameniinae	*Amenia* sp.	SRR6724164
			*Eurychaeta muscaria*	SRR6724165
			*Silbomyia hoeneana*	SRR11434663
		Bengaliinae	*Bengalia* sp.	SRR11434662
			*Verticia nigra*	SRR6724159
		Calliphorinae	*Calliphora vomitoria*	SRR1695328
			*Melinda viridicyanea*	SRR11434658
			*Polleniopsis* sp.	SRR11434659
		Chrysomyinae	*Chrysomya rufifacies*	SRR500993
			*Chrysomya megacephala*	SRR620248
			*Cochliomyia hominivorax*	SRR1532687
			*Protocalliphora* sp.	SRR11434661
			*Protophormia terraenovae*	DRR087979
		Luciliinae	*Hypopygiopsis tumrasvini*	SRR11434660
			*Lucilia cuprina*	GCA_001187945.1
		Phumosiinae	*Phumosia chukanella*	SRR11434657
		Rhiniinae	*Stomorhina subapicalis*	SRR1695394
		Rhinophorinae	*Bixinia* sp.	SRR6724163
			***Nesodexia corsicana***	SRR20824209
			*Stevenia* sp.	SRR6724161
	Mesembrinellidae		*Mesembrinella bellardiana*	SRR6724170
	Polleniidae		*Pollenia* sp.	SRR8236845
	Mystacinobiidae		*Mystacinobia zelandica*	SRR6724158
	Oestridae	Cuterebrinae	*Cuterebra austeni*	SRR1695306
	Sarcophagidae	Miltogramminae	*Miltogramma oestraceum*	SRR10753909
		Paramacronychiinae	*Agria mihalyii*	SRR10753923
		Sarcophaginae	*Sarcophaga carnaria*	SRR10753913
	Tachinidae	Phasiinae	*Gymnosoma nitens*	SRR6724168
		Exoristinae	*Pseudogonia rufifrons*	SRR6724155
	Ulurumyiidae		*Ulurumyia macalpinei*	SRR6724160

### Morphology

#### Adults

High-resolution photographs and measurements were captured using a Zeiss Axio Zoom V16 equipped with an Axiocam 208 color camera. The focus stacking software, Helicon Focus 8.2.2, allowed for the integration of photographs from various focal planes into comprehensive composite images. The dissection of male terminalia followed the methodology described by Cerretti and Shima (2011). Morphological terminology followed the guidelines outlined by Cumming and Wood (2017). Measurements of thoracic width (i.e., inter-tegular distance) and overall body length in *N. corsicana* were compared against *Paykullia insularis* (Villeneuve), *P. nubilipennis* (Loew), *P. partenopea* (Rondani), *Phyto adolescens* Rondani, *P. cingulata* (Zetterstedt), *P. melanocephala* (Meigen), *Phyto* sp. [cf. *cingulata*], *Rhinomorinia sarcophagina* (Schiner), *Stevenia atramentaria* (Meigen), *S. deceptoria* (Loew), *S. etrusca* Cerretti and Pape, *S. obscuripennis* (Loew), *S. palermitana* Cerretti and Pape, *S. signata* (Mik) and *Tricogena rubricosa* (Meigen) (Supplementary File S2). Redescription and measurements are based on pinned and alcohol preserved specimens as listed in “Material examined” below.

#### Eggs

Preparation for SEM involved dehydration through 99.9% ethanol and soaking for 2 × 30 minutes in hexamethyldisilazane (HMDS), followed by air-drying under a fume hood. Images were produced with a PHENOM PRO X SEM (Thermo Fisher Scientific Inc.) in the Department of Invertebrate Zoology and Hydrobiology, University of Lódź. Terminology follows Hinton (1981) with modifications proposed by Grzywacz et al. (2012).

### Acronyms of Collections Cited

IRSNB: Institut Royal des Sciences Naturelles de Belgique, Bruxelles [Brussels], Belgium.
MNHN: Muséum National d’Histoire Naturelle, Paris, France.
MZUR: Museum of Zoology, Sapienza Università di Roma, Rome, Italy.
NHMD: Natural History Museum of Denmark, Copenhagen, Denmark.
SMNS: Staatliches Museum für Naturkunde, Stuttgart, Germany.

### Red List Category and Potential Key Biodiversity Area (KBA) Assessment

An IUCN Red List category assessment for *N. corsicana* was performed by applying criterion B1 and B2 of the IUCN Red List criteria, which refer to the geographic range of a species (IUCN Standards and Petitions Committee, 2022). We calculated the Extent of occurrence (EOO) and AOO following the procedure reported in the Mapping Standards and Data Quality for the IUCN Red List Categories and Criteria (IUCN, 2021). A decline in the form of habitat reduction within the range of the species was estimated through a comparison between the Area of Habitat (AOH) map and the species’ occurrence points. This information was needed for a correct application of criterion B2 (IUCN Standards and Petitions Committee, 2022).

The AOH map shows the distribution of the habitat considered to be available to the species within its geographic range and under the assessed elevation limits (Nania et al. 2022). We produced an AOH map using the EOO to delineate the boundaries of the species’ geographic distribution. We then implemented the high-resolution (100 m) CLC2018 land cover map for Corsica produced by the European Commission’s CORINE program, using its 44 land cover categories as a proxy to map the habitat of the species within its EOO. Elevation data were retrieved from the Shuttle Radar Topography Mission (Earth Resources Observation and Science (EROS) Center 2019). Information on the species’ habitat requirements and elevation range is based on the observations retrieved from all available occurrence points; the information is available in Supplementary File S3. AOH maps have already been produced for other taxa following a similar method (Lumbierres et al. 2022, Nania et al. 2022, 2024). The AOH map was validated using hypergeometric distribution approach (Dahal et al. 2022). Details of the validation test are available in Supplementary File S3. Additionally, we produced a second AOH map following the same procedure described above, but instead of limiting the habitat mapping to the species’ EOO, we mapped the habitat within the geographic boundaries of the island. The second AOH map was used to estimate the potential distribution of the species on the island of Corsica beyond the currently known localities.

We performed a scoping analysis of potential KBAs for criterion A1 (threatened species) and B1 (geographically restricted species). Both criteria can be applied using the AOH map alone to estimate the relative percentage of the global population size of the species within a potential KBA site (IUCN, 2016). We adopted the systematic approach for scoping potential KBAs presented by Nania et al. (2023) and have already implemented it on insect data (Nania et al. 2024). The method uses a 10 × 10 km cell grid to scan the geographic surface, delimited by the species’ geographic range and identifies sites that hold a percentage of the global population of the species within them that is sufficient to trigger a potential KBA under the tested criteria. The resulting potential KBAs map has the same resolution of the CLC2018 map. Potential KBA boundaries are defined by the extent and distribution of habitat that was able to trigger a potential KBA within a grid cell. We assessed the percentage of new potential KBAs that are already included in national protected areas by calculating the total extent of potential KBAs falling within the boundaries of the regional park of Corsica. A map of the regional park of Corsica was retrieved from the open data platform https://www.data.gouv.fr, developed by the French Ministry of Public Sector Transformation and the Civil Service (https://www.data.gouv.fr/).

## Results

### Phylogenetic Reconstruction

Genome skimming data generated for *Nesodexia* assembled into 515,807 contigs of which 119,986 contigs with coverages > 10× were included for downstream analysis. Orthology prediction detected 2,665 single-copy genes.

The concatenated single-copy protein-encoding supermatrix yielded 3,196 single-copy protein-encoding genes after all postprocessing steps (alignment, alignment refinement, outlier check and outlier removal, identification and deleting ambiguously aligned sections from the MSAs, and corresponding processes on nucleotide level) and the gene occurrence of this supermatrix was 64.3% (Information Content = 0.393). Post MARE optimization improved the gene occurrence of the supermatrix to be 87.3% (IC = 0.591), and the final dataset consists of 1,674 single-copy protein-encoding genes.

**Fig. 1.**
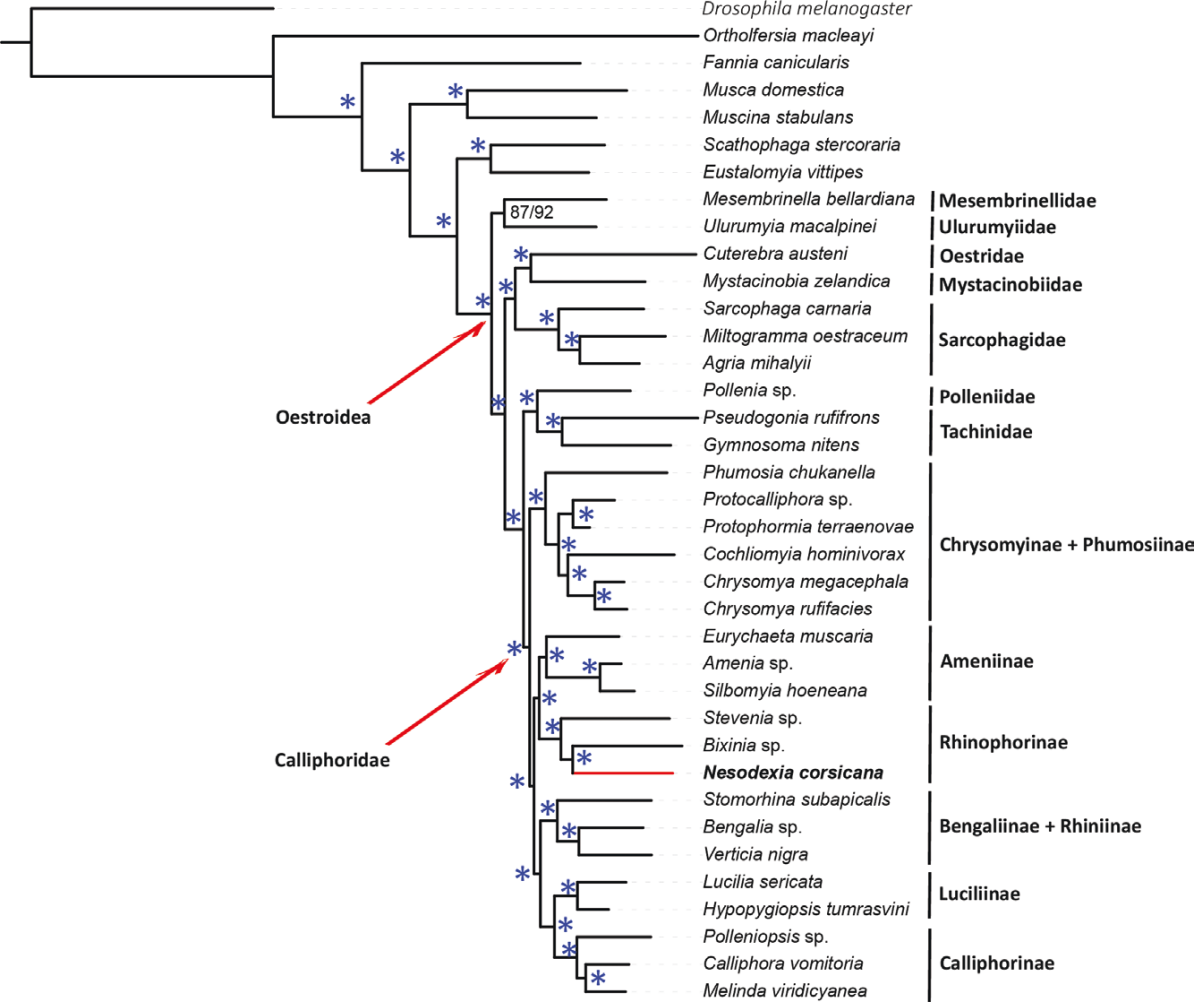
ML tree of Oestroidea (Diptera) inferred using IQTREE version 2.0.5, based on AA and NT12 matrices. Numbers at branches indicate ultrafast bootstrap resampling values of phylogeny reconstruction using the matrices AA/NT12. The asterisk (*) indicates full support.

The resulting calyptrate phylogenetic tree has a representation of every oestroid family and most of the currently accepted subfamilies (Table 1). All branches have received full support, with the sole exception of the branch below the clade Ulurumyiidae + Mesembrinellidae. *Nesodexia corsicana* emerges inside the blow flies subfamily Rhinophorinae, being sister to one of the two included exemplar species (Fig. 1).

### Systematics Order: Diptera Suborder: Brachycera Family: Calliphoridae, subfamily: Rhinophorinae Genus *Nesodexia*Villeneuve, 1911

*Nesodexia* Villeneuve, 1911: 123 [original description]. Type species: *Nesodexia corsicana*Villeneuve, 1911: 123, by original designation.

#### Originally Included Species

*Nesodexia corsicana* Villeneuve, 1911.

#### Type Material

1 male, 1 female syntypes (SMNS) labeled: “Campo di Loro (Corse)”; 2 male syntypes (NHMD) labeled “Campo di Loro (Corse) 22-vi,” one with an additional label “*Nesodexia corsicana* type Villen.” In Villeneuve’s hand. Other male and female syntypes are preserved at IRSNB.

#### References


Cerretti et al. (2019), Gisondi et al. (2020), Mesnil (1980), Pape et al. (2015), Peris and González-Mora (2004), Rognes (1991, 1997, 1998, 2014), Schumann (1986), Séguy (1928, 1941), Townsend (1938), Zumpt (1956).

#### Diagnosis

**Fig. 2.**
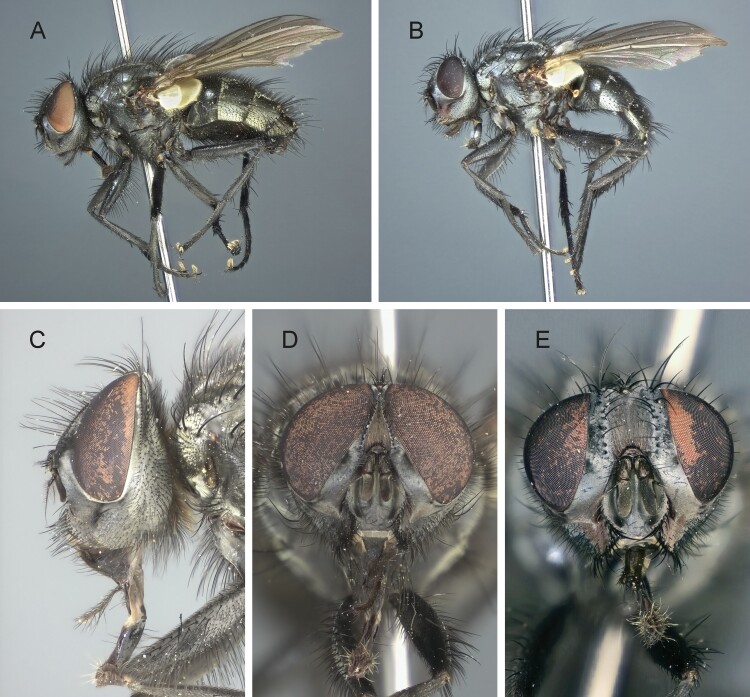
*Nesodexia corsicana* Villeneuve (Calliphoridae: Rhinophorinae). A and B) Habitus in lateral view: A) male, B) female, C) male head in lateral view, and D and E) head in frontal view: D) male, E) female.

**Fig. 3.**
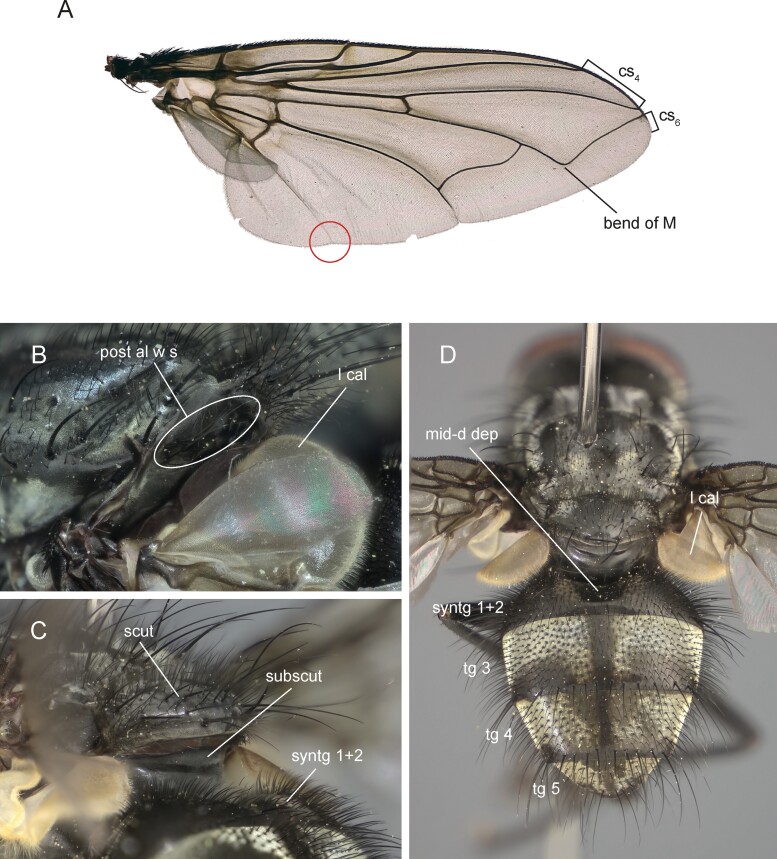
*Nesodexia corsicana* Villeneuve (Calliphoridae: Rhinophorinae). A) right wing, male [circle indicates vein CuA + CuP reaching wing margin], B) detail of left side of thorax in dorsolateral view, C) detail of part of thorax and abdomen in posterolateral view, and D) male habitus in posterodorsal view. Abbreviations: cs4—costal sector 4; cs6—costal sector 6; l cal—lower calypter; mid-d dep—mid-dorsale depression; post al w s—postalar wall setae; scut—scutellum; subscut—subscutellum; syntg 1+2—syntergite 1+2; tg3-5—tergites 3 to 5.

**Fig. 4.**
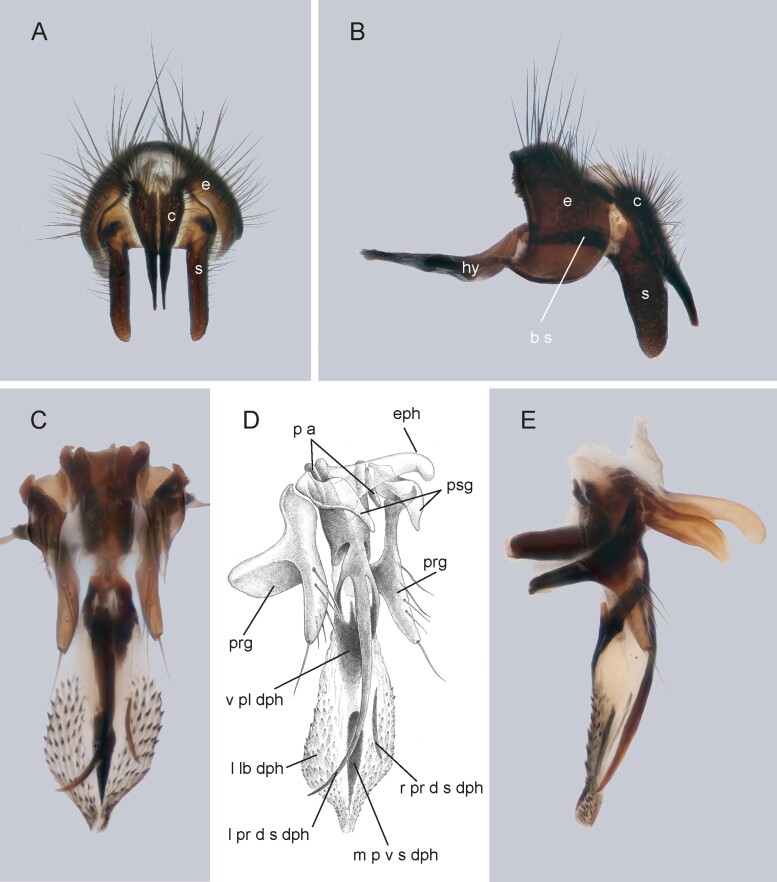
Male terminalia of *Nesodexia corsicana* Villeneuve (Calliphoridae: Rhinophorinae). A and B) epandrian complex: A) posterior view, B) lateral view; (C–E) phallus: C) posterior view, D) posterolateral view, E) lateral view. Drawing by: M. Mei. Abbreviations: b s—bacilliform sclerite; c—cercus; e—epandrium; eph—epiphallus; hy—hypandrium; l lb dph—lateral lobe of distiphallus; l pr d s dph—left process of dorsal sclerite of distiphallus; m p v s dph—median process of ventral sclerite of distiphallus; p a—postgonal apodeme; prg—pregonite; psg—postgonite; r pr d s dph—right process of dorsal sclerite of distiphallus; s—surstylus; v pl dph—ventral plate of distiphallus.

Medium sized blowfly, dark grayish in general appearance. Living specimens are very reminiscent of *Pollenia* specimens in the way they rest on leaves, flowers or on the ground, with wings slightly spread and body resting on the substrate (i.e., not raised on legs). Head profile not receding. Parafacial bare. Proepisternum bare. Anatergite and postalar wall with tuft of setulae. Anterior and posterior fringes of posterior spiracle unequal in size: posterior lappet distinctly larger. Lower calypter broad (Fig. 2A and B; Fig. 3B and D), that is, not tongue-shaped as typical in the rhinophorine. Hind tibia with three preapical dorsal setae. Preapical posteroventral seta of hind tibia about as long as preapical anteroventral seta. Subscutellum weakly convex. Dorsal sclerite of distiphallus asymmetrical: left process of dorsal extension long, blade-like with tip free from phallic wall; right process reduced to small, narrow sclerite lying on phallus membrane, not fused with dorsal sclerite (Fig. 4C and D). Median process of ventral sclerotization of distiphallus not fused with ventral plate of distiphallus.

#### Autapomorphies

Dorsal sclerite of distiphallus asymmetrical: right process of dorsal extension reduced to small, narrow sclerite separated from remaining dorsal sclerite (Fig. 4C and D).

### *Nesodexia corsicana* Villeneuve

#### Material Examined

1 male: France—Corsica: Haute-Corse, Sisco, 42°48’N; 09°26’E, 25-300 m, 2.VII.2021, P. Cerretti leg. [NHMD]. 1 male: France—Corsica: Haute-Corse; Chioso. 42.818467° N; 9.434145° E; 225 m. 2.VII.2021; P. Cerretti leg. [MZUR]. 20 males, 5 females: France—Corsica: Corse-du-Sud, D55, Cognocoli-Monticchi, 41°51’04.4’‘N, 08°54’00.8’‘E, 730m, 4.VII.2021-7.VII.2021, P. Cerretti leg. [MZUR and NHMD]. 2 females: France—Corsica: Porto. 10 km E Porto [decLat 42.2619, decLon 8.8291], 8.VII.1967; Langemark-Lomholdt leg. [NHMD]. 1 male: France—Corsica: Zonza, Samulaghia, marshy seep in dry Sapinière forest, 41°45’39.6‘N, 9°13’37.2’E, 1244m, 24–28.vi.2019, Marc Pollet leg. [MNHN]. 1 female: France—Corsica: Zonza, Samulaghia, on rocky seep in Sapinière forest (edge of forest), 41°45’40.1”N, 9°13’32.9”E, 1231 m, 24–28.vi.2019, Marc Pollet leg. [MNHN].

#### Diagnosis

As for the genus.

#### Redescription

**Fig. 5.**
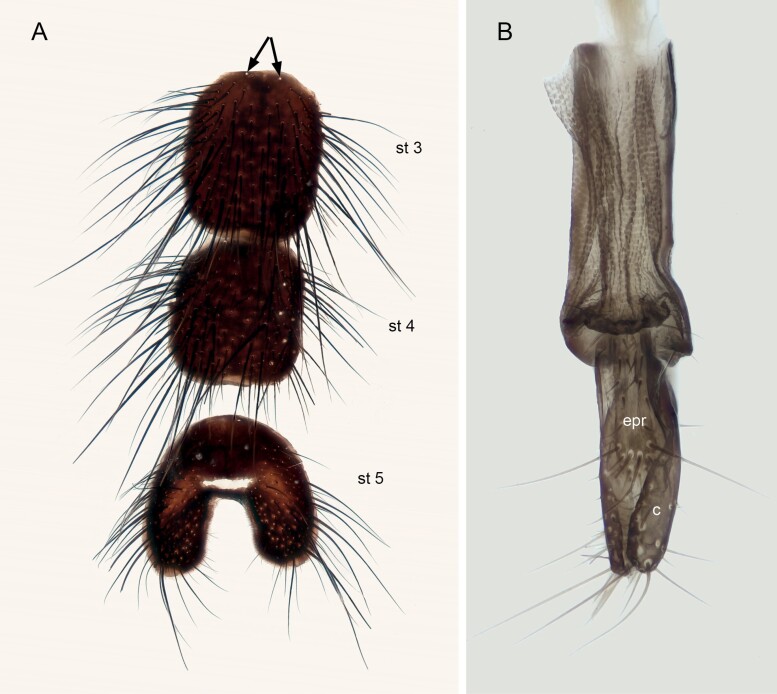
*Nesodexia corsicana* Villeneuve (Calliphoridae: Rhinophorinae). A) Male abdominal sternites 3–5 [arrows indicate sockets for sensilla trichodea or “alpha-setae”], B) female oviscapt. Abbreviations: c—cercus; epr—epiproct; st 3-5—strenite 3 to 5.

Male (Fig. 2A, C, and D; Fig. 3A–D; Fig. 4A–E; Fig. 5A).

Body length (a): 7.5–10.1 mm; distance between tegulae (b): 2.4–3.7 mm; b/a = 0.31–0.37 [mean: 0.33].

##### Color.

Head black in ground color covered with thin, silvery gray microtomentum; frontal vitta brownish; area between gena and parafacial dark brown; scape, pedicel, and postpedicel uniformly dark brown; palpus blackish. Occiput and genal dilation with only black setulae. Thorax black in ground color, mostly covered with silvery gray microtomentum, with three broad pre- and postsutural dark vittae in posterior view, the central vitta flanked by two thinner, less evident ones. Scutellum black, with two lateral silvery spots of microtomentum. Legs black. Upper and lower calypters whitish; wing membrane almost hyaline, infuscated only in the proximal part of bc, c and br cells; tegula and basicosta black; veins dark brown to black; halter yellowish. Abdomen black in ground color; syntergite 1 + 2 with thin spots of silvery gray microtomentum laterally on both sides of mid-dorsal depression; tergites 3–4 each with a wide band of microtomentum, broadly concave posteriorly and medially interrupted by a bare longitudinal stripe.

##### Head

(Fig. 2A, C, and D). Nearly holoptic, frons about 1/8 of a compound eye in dorsal view (Fig. 2D). Face not receding. Inner vertical setae is well developed, approx. 0.5× as long as compound eye height crossed medially. Outer vertical seta barely differentiated from postocular setulae. Ocellar triangle with two pairs of proclinate ocellar setae and 2–3 pairs of shorter proclinate setulae. Frons with 10–12 frontal setae descending to the upper margin of the scape. Fronto-orbital plate with minute setulae on distal half. Upper reclinate orbital setae absent. Proclinate orbital setae absent. Parafacial bare, approximately 1.5–1.6× as wide as postpedicel at mid-length. Facial ridge concave with several short and fine to long and stout setae above vibrissa, on lower 1/3 or slightly more. Vibrissa inserted above the level of the lower facial margin. Face not visible in lateral view in front of vibrissal triangle; lower facial margin slightly visible in profile. Gena approximately 0.28–0.30 of compound eye height. Genal dilation is well developed. Antenna about as long as the height of gena, or slightly shorter. Postpedicel 2.0–2.2× as long as pedicel. Arista thickened on proximal 1/5, with long trichia 3–4× longer than its greatest diameter. First aristomere very short, much wider than long. Second aristomere longer, 1.2 as long as wide. Prementum about 3× as long as its width at mid-length. Palpus apically hardly enlarged.

##### Thorax

(Fig. 3B and C). Proepisternum bare. Postpronotum with 3–6 setae arranged in a triangle. 2–3 presutural and 2–4 postsutural acrostichal setae. Three presutural and 4 postsutural dorsocentral setae. One presutural and three postsutural intra-alar setae. First postsutural supra-alar seta is well developed, that is, about as long as notopleural setae or slightly longer. Three to 5 katepisternal setae. Katepimeron with fine hair-like setulae on anterior fourth. Anepimeral not differentiated. One pair of strong apical scutellar setae, crossed. 1–2 pairs of subapical and 1–2 pairs of basal scutellar setae. Anatergite and postalar wall with tuft of setulae (Fig. 3B). Subscutellum weakly convex (Fig. 3C). Anterior and posterior fringes of posterior spiracle unequal in size: posterior lappet distinctly larger. *Legs*. Preapical anterodorsal seta of fore tibia about as long as preapical dorsal seta. Mid tibia with 1–2 anterodorsal seta. Hind tibia with three preapical dorsal setae. Preapical posteroventral seta of hind tibia about as long as preapical anteroventral seta. Hind tibia with 4–6 well-developed anterodorsal setae. Posterodorsal margin of hind coxa bare. *Wing* (Fig. 3A). Lower calypter broad (Fig. 2A and B; Fig. 3B and D). Wing membrane is mostly hyaline except occasional brownish shading at the base and along veins in some specimens. Second costal section (cs_2_) bare ventrally. Costal spine not differentiated. R_1_ bare. Base of vein R_4 + 5_ with 1–4 setulae dorsally and ventrally, occasionally bare. Bend of M forming obtuse angle. Fourth costal section (cs_4_) longer than sixth (cs_6_). Section of M between crossveins r-m about 2.0–2.5 times as long as section of vein between dm-m and bend of M. Cell r_4 + 5_ open. Vein CuA + CuP reaching wing margin (Fig. 3A, red circle).

##### Abdomen

(Fig. 3D). Mid-dorsal depression of syntergite 1 + 2 confined to anterior 7/8 of syntergite. Syntergite 1 + 2 without median marginal setae. Tergite 3, 4, and 5 with a row of marginal setae. Tergite 5 short, approximately 2/5 as long as tergite 4. Abdominal sternites exposed; sternites 3 and 4 with a pair of alpha setae on anterior margin. *Terminalia* (Fig. 4A–E; Fig. 5A). Sternite 5 with deep, wide, posteromedian notch with narrow membranous window (Fig. 5A). Transversal section of sternite 5 shallowly U-shaped. Tergite 6 broad, with a narrow indentation antero-medially with several fine setulae. Connection between tergite 6 and syntergosternite 7 + 8 membranous. Connection between sternite 6 and syntergosternite 7 + 8 membranous on the right side. Epandrium very short and convex; anterior extension is well developed, posterolateral lobe scarcely developed. Cerci normally developed, basally wide, not fused medially at the base (i.e., suture between cerci complete and visible). Surstylus normally developed, basally wide, narrowing distally (distal third lobe-like in lateral view); setae on median extension present. Connection between bacilliform sclerite and antero-basal portion of surstylus membranous. Connection between surstylus and epandrium membranous. Median plate of hypandrium short, flat, and narrow, hypandrial arms very long, narrow, and subparallel. Phallic guide broad, bilobed. Postgonal apodeme is well developed. Connection between phallic guide and pregonite membranous (i.e., not fused). Pregonite is well developed, distal lobe subtriangular in lateral view with a row of sensilla along its posterior margin and a long sensillum at tip. Anterior seta on postgonite absent. Dorsal sclerite of distiphallus asymmetrical: left process of dorsal extension long, blade-like with tip free from phallic wall; right process reduced to small, narrow sclerite lying on phallus membrane, not fused with dorsal sclerite (Fig. 4C and D). Median process of ventral sclerotization of distiphallus not fused with ventral plate of distiphallus (Fig. 4C–E). Median process of ventral sclerotization of distiphallus longitudinally not divided. Lateral lobes of distiphallus broad with long, feather-like, sclerotized spines. Acrophallus simple (i.e., with one opening).

##### Female

(Fig. 2B and E; Fig. 5B). Differs from males as follows: outer vertical seta present, well developed; one pair of upper lateroclinate orbital setae; two pairs of proclinate orbital setae; 1 pair of lateroclinate postocellar setae; frons about 3/4–4/5 of a compound eye in dorsal view. *Terminalia* (Fig. 5B). Oviscapt moderately long and telescopic, retracted in fifth segment. Segments 7 and 8 normally developed and unmodified. Tergite 8 rectangular and cerci narrow, normally developed, straight. Three suboval spermathecae, dark brown in color.

##### Egg

**Fig. 6.**
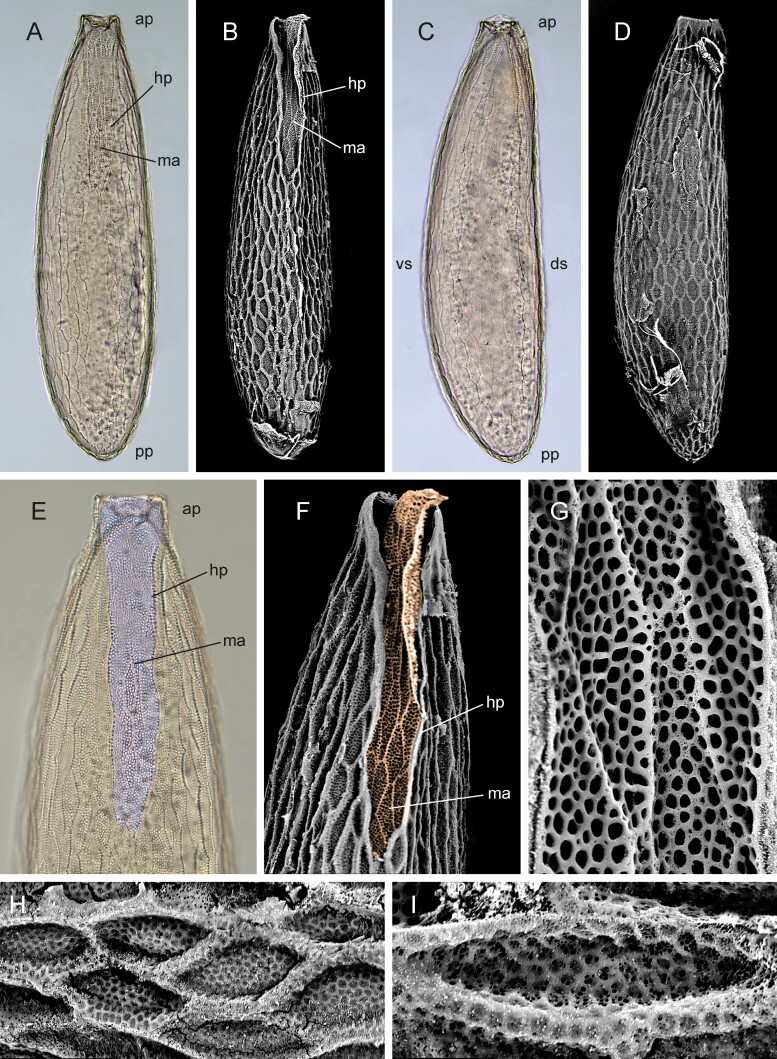
Egg of *Nesodexia corsicana* Villeneuve (Calliphoridae: Rhinophorinae). A and B) Dorsal view: A) light microscope, B) SEM; C and D) lateral view: C) light microscope, D) SEM; E and F) anterior third in dorsal view: E) light microscope, F) SEM; G and H) SEM images: G) detail of plastron, H–I) details of chorion. Abbreviations: ap—anterior pole; ds—dorsal surface; hp—hatching pleats; ma—median area; pp—posterior pole; vs—ventral surface. Color coding: purple—plastron.

(Fig. 6). Eggs are white in color, elongated, oval in cross-section, small in size, with a length of 0.63–0.65 mm, and a width of 0.17–0.18 mm (*n* = 10). Anterior pole truncate, posterior pole rounded (Fig. 5A–F). Ventral surface convex, dorsal surface flat to slightly concave with broad median area and lateral fold-like hatching pleats. Hatching pleats converge posteriorly and meet at one-third of egg length (Fig. 6A–D). Plastron of median area covered with extensively perforated hexagons (Fig. 6E and F). The surface of the hexagons forming regular perforations (Fig. 6G). The chorion of the entire remaining surface of the egg is covered with elongated hexagons parallel to the longitudinal axis of the egg (Fig. 6H and I). Hexagons in this area with reticulation, each mesh of the net with several small perforations (Fig. 6H and I).

##### Larva.

Unknown.

### Red List Category and New Potential KBAs

The Red List category assessment resulted in the evaluation of *N. corsicana* as ‘endangered’ under criteria B1 and B2ab. The total EOO of the species is equal to 3,012 km^2^. A species can be assessed as “endangered” under criterion B1 if its EOO is ≤ 5,000 km^2^. The total AOO calculated based on all currently available occurrence points is equal to 24 km^2^, which is below the threshold of 500 km^2^ reported in the IUCN Red List guidelines for a species to be assessed as “endangered” under criterion B2. The results of subcriterion B2a (fragmentation and number of localities) are consistent with the “endangered” category, matching the threshold of 5 clearly distinct localities. The AOH map was evaluated as better than a random model, with only one record falling out of the mapped habitat (Supplementary File S3). A comparison between the AOH map and the occurrence points highlighted that the type locality occurrence, recorded in 1907, is now the only point not falling within the mapped habitat of the species, revealing a possible loss of habitat extent overtime. This is consistent with subcriterion B2b (continuing decline observed in extent of habitat) (IUCN, 2022).

**Fig. 7.**
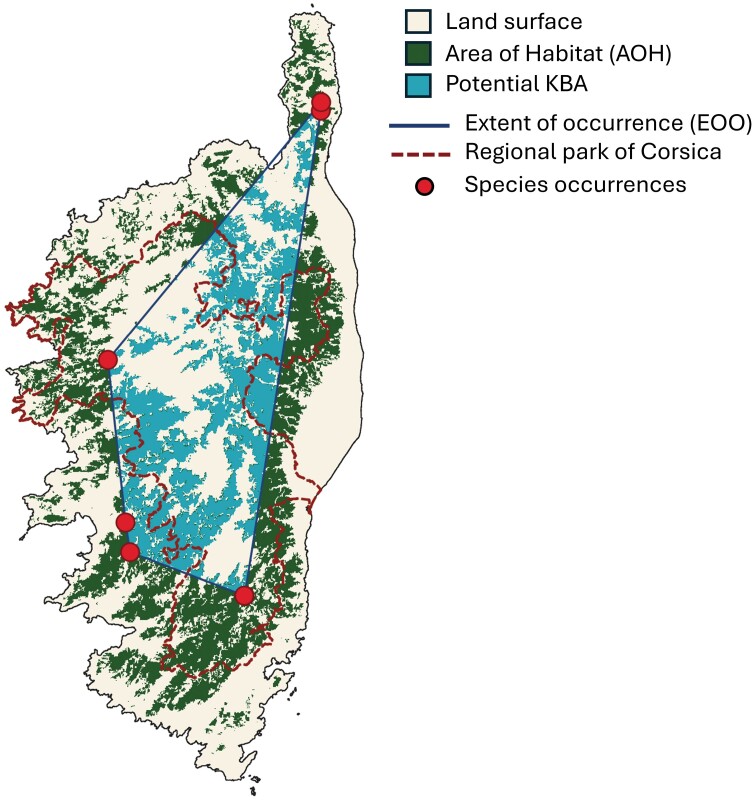
The map shows the extent of habitat mapped inside and outside of the EOO of the *Nesodexia corsicana*. The identified potential KBAs are shown to be only partially located within protected area boundaries, delimited by the regional park of Corsica.

The potential KBA assessment revealed that 99.7% of the AOH corresponds to potential KBAs under criterion A1 (Fig. 7). The total area of potential KBAs is 1,443 km^2^. The scoping analysis did not detect potential KBAs under criterion B1. The potential distribution of the species across the island, based on the AOH, is illustrated in Fig. 7. The total area extent of the habitat across the island is 3,559 km^2^. The percentage of new potential KBAs already encompassed by the regional park of Corsica was found to be 60%.

## Discussion

The vast production of genomic data and the parallel development of refined analytical methods are instrumental in delineating an increasingly detailed picture of the phylogenetic relationships between living organisms. A robust phylogenetic reconstruction makes it possible to formulate explicit hypotheses on the evolution of life at various levels, the functioning of ecosystems in time and space, as well as revealing relationships that had remained elusive because they were hidden by sometimes surprisingly indecipherable phenotypes (Baker et al. 2016, Giribet and Edgecombe 2020). Robust phylogenies also facilitate stable, coherent, informative, and predictive classification schemes, which allow them to be used at different levels of application, such as bioprospecting and nature conservation (Faith 2018, Lüddecke et al. 2019). In recent years, phylogenetic relationships within Oestroidea have been studied by several research groups using different taxon samples and data, from Sanger sequences (Kutty et al. 2010, Singh and Wells 2013, Cerretti et al. 2017) to phylogenomics (Kutty et al. 2019, Buenaventura et al. 2020, Yan et al. 2021). These studies have shown progressive agreement over time in reconstructing phylogenetic relationships within the group, and the large amount of data produced so far has made it possible to resolve many long-standing questions and shed further light on the multitude of evolutionary trajectories within this clade (Blaschke et al. 2018, Stireman et al. 2019, 2021, Yan et al. 2019, 2021, Li et al. 2020).

### *Nesodexia corsicana* in the Oestroidea Tree of Life

As expected, the addition of *N. corsicana* did not alter the phylogenetic relationships between family and subfamily ranked clades with respect to the cladogram obtained by Yan et al. (2021), returning maximum support values at all nodes, except for the monophyly of the clade formed by *Ulurumyia* and *Mesembrinella* which showed moderate support values (Fig. 1), consistent with other studies (Cerretti et al. 2017, Kutty et al. 2019, Yan et al. 2021). Contrary to expectations (see Rognes 1991, 1997, 1998), *N. corsicana* did not group with the Polleniidae but turned out nested within the Rhinophorinae, a clade of Calliphoridae traditionally treated as a family (Crosskey 1977, Cerretti et al. 2020) and suspected to be exclusively parasitoids of woodlice (Malacostraca: Oniscidea) (Bedding 1965). Life history data for the Rhinophorinae are limited to a handful of species (Bedding 1973, Wood et al. 2018, Cerretti et al. 2020), but the phylogenetic distribution of the taxa known to be wood lice parasitoids is such that it can be assumed that the exploitation of oniscideans represents the ancestral state of rhinophorines (Pape 1986, Cerretti et al. 2014, 2020); shifts to other hosts by some lineages are not excluded, although here considered unlikely.

### Misleading Morphology

As noted above, the history of *Nesodexia* classification has been troubled, and only Rognes (1997) has addressed its phylogenetic position using cladistic arguments. While stressing the need for further investigation, Rognes (1991, 1997) argued in favor of *Nesodexia* being placed in the Polleniinae (now Polleniidae) by recognizing three synapomorphies, all of which showed extensive homoplasy: position of outer posthumeral seta in line with the presutural seta, two spermathecal ducts joined shortly before connecting with the common oviduct, and a pair of projections (lingulae) directed anteriorly from the base of the female hypoproct. *Nesodexia* also shares other character states (now known to be either plesiomorphies or homoplastic apomorphies) with many species of *Pollenia*, such as a superficial similarity in body size, shape, and coloration (being extensively covered with gray reflecting microtomentum), circular metathoracic spiracles of moderate size, and anterior and posterior spiracular fringes unequal in size (ground plan trait of oestroid flies). In addition, resting *Nesodexia* specimens hold their wings slightly apart and can be easily mistaken for a polleniid or a tachinid in the field. Rognes (1997, Fig. 2A) reconstructs the ‘*Nesodexia* + Polleniinae’ clade, nested within a large clade comprising almost all subfamilies of the then family Calliphoridae (with the sole exception of Rhiniinae), based on three synapomorphies, namely (i) broad lower calypter, (ii) postalar wall with setae, and (iii) third instar larva with parastomal bars. This clade was, in turn, reconstructed as the sister group of the Rhinophorinae (then given family rank). In the cladistic analyses of Pape (1992), Rognes (1997) and Cerretti et al. (2017), the Rhinophorinae were never recovered within the Calliphoridae.

All recent phylogenetic reconstructions of oestroid flies (Cerretti et al. 2019, Kutty et al. 2019, Yan et al. 2021, Gisondi et al. 2023) converge on topologies supporting that the shape, either wide or narrow, of the lower calypter, the size and shape of the metathoracic spiracular fringes, and the presence/absence of setae on the postalar wall are likely determined by multiple and independent evolutionary pathways of gains and losses, as these characters are highly homoplastic when optimized over these trees. For example, the lower calypter of many Polleniidae (*Alvamaja*, *Melanodexia*, *Morinia*) are narrow and tongue-shaped, as in rhinophorine and rhiniine calliphorids. Similarly, the anterior and posterior fringes of the metathoracic spiracle of *Alvamaja*, *Melanodexia,* and *Morinia* are small, outwardly directed and of equal size, as in virtually all rhinophorines (*Baniassa* Kugler and *Maurinophora* Cerretti and Pape are notable exceptions) and several tachinids (Crosskey 1977, Cerretti et al. 2014, 2020). Cerretti et al. (2019) showed that such similarities have strongly contributed to obscuring the phylogenetic relationships of *Alvamaja* and several *Morinia*, which were originally assigned to Rhinophorinae. The same applies to *Nesodexia*, where its wide lower calypters and setulose postalar wall, neither of which are shared with any known rhinophorine, have consistently misled taxonomists with regard to its classification.

### Could Any Clues Have Led to the Right Track?

Despite being separated by a considerable phylogenetic distance, Polleniidae and Rhinophorinae have often been confused, and *Nesodexia* is apparently just another case where certain morphological features can be misleading. However, some clues must have been picked up by Benno Herting—expert taxonomist, and specialist on Tachinidae who served as Diptera curator at the Staatliches Museum für Naturkunde Stuttgart (SMNS) from 1969 to 1988 (Tschorsnig 2005)—but were never published. Taxonomists have traditionally conveyed information through publications and through the organization of their curated collections. Museum curators use various methods to arrange taxa, such as alphabetically by genus and species, according to a taxonomic catalogue, or based on inferred relationships in a “work in progress” style. Typically, specimens with uncertain affinities are placed at the end of a genus or family. For example, at SMNS, questionable Diptera specimens have historically been organized according to the last-named species in their respective genus or family. According to H.-P. Tschorsnig (former Diptera curator at SMNS), Benno Herting was responsible for curating the rhinophorine collection since the late 1960s, and he deliberately placed the museum’s two specimens of *N. corsicana* (see *Materials and Methods* section) in the last drawer of identified rhinophorines (https://ent.smns-bw.org/drawer/Diptera/Rhinophoridae/Rhino_004.html). We do not know when Herting had this possible Darwinian “I think...” moment about the possible affinities of *Nesodexia*, but we are happy to validate his opinion and to provide this testament to his deep understanding of rhinophorine relationships.

### Morphological Support for *Nesodexia* as Rhinophorinae and Its Autapomorphies

In light of our results, certain morphological features of *Nesodexia* provide interesting insights. Some important character states shared with other rhinophorines are: (i) occiput with only black setulae (seemingly yellowish setulae result from misleading light reflections), (ii) median extension of surstylus with setae, and (iii) median process of ventral sclerotization of distiphallus not fused to ventral plate of distiphallus. Pale occipital setulae characterize many oestroids and may represent a ground plan apomorphy of this superfamily; however, exceptions are widespread throughout the clade and found in almost every family. The two features from the male terminalia also occur in other oestroid lineages, mostly Tachinidae and Calliphoridae, but with the rhinophorines both are uniquely shared with genus *Phyto* and members of the tribe Phytonini (Cerretti et al. 2020, Gisondi et al. 2023). These three features, and especially the last two, are so far the only character states we have been able to find that provide morphological support for placing *Nesodexia* as a member of the Rhinophorinae.

In addition to the unique combination of character states just discussed, *Nesodexia* has a conspicuous, unambiguous autapomorphy: the dorsal extension of the phallus is asymmetrical, with the right process being reduced to a small, narrow sclerite that is not fused with the dorsal sclerite (Fig. 4C and D). Asymmetries in male genitalia are widespread in animals, and their occurrence in otherwise bilateral organisms by itself asks for explanation (Schilthuizen 2013). Asymmetrical male genitalia are common in Diptera (Huber et al. 2007), but they are rare events in the oestroid flies. Asymmetry involving the distiphallus is known from a few tachinids, especially among the Phasiinae (Tschorsnig 1985, Tschorsnig and Richter 1998, Blaschke et al. 2018), in the Sarcophagidae from a few species of *Oxysarcodexia* Townsend and from *Sarcophaga princeps* (Wiedemann) (Sugiyama et al. 1990, Souza et al. 2014), and in the Calliphoridae from some species of the rhinophorine genus *Ventrops* Crosskey (Cerretti and Pape 2012, Cerretti et al. 2015, 2020). The phallic asymmetry in *Nesodexia* differs from all these cases by involving the dorsal extension.

### Classification and Natural History Implications

The inclusion of only two (other) representatives of the Rhinophorinae in our analyses prevents us from making well-supported hypotheses regarding the fine phylogenetic affinities of *Nesodexia*. In addition to the features of the male terminalia mentioned as shared with members of Phytonini, *N. corsicana* shares with species of *Phyto* (i) a similar shape of the phallus, characterized by long, blade-like extensions of the dorsal sclerite of the distiphallus, the tips of which are free from the phallic wall, and by broad lateral lobes of the distiphallus, which are covered with feather-like, sclerotized spines, (ii) a broad, bilobed phallic guide (Tschorsnig 1985: Fig. 16), (iii) medially fused surstyli, and (iv) a well-developed first postsutural supra-alar seta. This combination of character states supports *Nesodexia* as a member of the Phytonini (see Gisondi et al. 2023).

The placement of *Nesodexia* within the rhinophorines raises an important question about its biology: Is it a parasitoid, and if so in which host? Egg morphology provides no specific clue, except that the unspecialized chorion and the presence of a well-developed plastron indicate the absence of female incubation and unspecialized oviposition. As such, *Nesodexia* is in agreement with most rhinophorines (Bedding 1973, Pape and Arnaud 2001, Draber-Mońko 1997). As mentioned earlier, while it cannot be ruled out that *Nesodexia* exhibits various peculiarities, including a significant change in host or developmental strategy, we find it more likely that this species develops as an endoparasite of Oniscidean Isopods. If so, which of the 76 Corsican oniscid species (Taiti and Ferrara 1996) possesses the characteristics to be a potential host for *Nesodexia*?

The size of the adult *Nesodexia* is noteworthy; it is the largest known rhinophorid (see Supplementary File S2). Although specimens in the genus *Paykullia*, *Phyto* and *Stevenia*, especially of *S. etrusca* Cerretti and Pape, may occasionally reach a maximum length of 10.7 mm, none have an average width (measured between the tegulae) of 2.98 mm and a ratio between the width (measured between the tegulae) and body length of 0.33 (Supplementary File S2). The large body size of this fly narrows the potential hosts down to a handful of woodlouse species large enough to support *Nesodexia* larvae. The largest recorded woodlice on the island are *Armadillidium assimile* Budde-Lund, *A. nasatum sardoum* Arcangeli, *A. sordidum* Dollfus, *A. vulgare* (Latreille), *Helleria brevicornis* Ebner, *Porcellio dilatatus* Brandt, *P. laevis* Latreille, *P. orarum vizzavonensis* Verhoeff and *Tiroloscia corsica* (Dollfus) (S. Taiti, pers. comm.). Of all these taxa, only *A. orarum vizzanovensis* is endemic to Corsica. *Tiroloscia corsica* is also recorded from Sardinia, and all the others have a wider distribution (Boyko et al. 2023). Most of them have a body length ranging from 10 to 15 mm, except *H. brevicornis*, which reaches 25–30 mm in length and may be the most likely target for *Nesodexia*. *Helleria brevicornis* has a Tyrrhenian distribution (southern France, Corsica, Sardinia, the Tuscan archipelago, and the Piombino promontory). The next step in understanding the natural history of this enigmatic species is to verify the actual parasitism and identify the specific host species involved.

### Relevance for Conservation

In light of the IUCN Red List and potential KBA assessment, *N. corsicana* was found to be highly relevant for biodiversity conservation, as it was assessed as endangered, and almost the totality of its AOH was evaluated as potential KBA. The assessment is based on all available occurrence points and the full knowledge of the species’ distribution as it is currently known. However, the AOH map built across the entire island reveals a possible broader distribution of the species. The lack of occurrence data outside of the EOO may be due to incomplete sampling, and not necessarily imply the absence of the species. This is a well-known issue in IUCN Red List assessments of invertebrate species that can lead to overestimation of the risk of extinction (Cardoso et al. 2011). This aspect further underlines the necessity of assembling and sharing insect occurrence datasets to improve such assessments.

Parasitoid flies such as *N. corsicana* play a crucial ecological role in terrestrial food webs by controlling and regulating their host populations. The loss of parasitoid species can disrupt ecosystem equilibrium; therefore, it is important to identify the host used by *N. corsicana* and the degree of trophic specialization. The size and different morphological features of this fly, together with its presumed association with sowbugs, suggest a high degree of specialization, implying an indirect dependence on the environmental preferences, abundance, and accessibility of its host.

Following the conservation assessment presented in this study, we recommend appropriate monitoring of this species in the future, focusing on its biology and distribution. The collection of new data on the species will allow the development of informed conservation measures, if necessary.

## Supplementary Material

ieae073_suppl_Supplementary_Files_S1

ieae073_suppl_Supplementary_Files_S2

ieae073_suppl_Supplementary_Files_S3
