# Supplementary material for: Phylogenomics resolves long-standing questions about the affinities of an endangered Corsican endemic fly
Source: J Insect Sci. 2024 Jul 25;24(4):9. doi: 10.1093/jisesa/ieae073 (PMC11271022; doi:10.1093/jisesa/ieae073)
Supplement: ieae073_suppl_Supplementary_Files_S1 [file ieae073_suppl_supplementary_files_s1.docx]

**Phylogenomics resolves long-standing questions about the affinities of an endangered Corsican endemic fly**

**Supplementary file 1. Orthologous sequence alignments**

Orthologs were summarised using custom *perl* scripts, internal stop codons as well as Selenocysteine, if any, were replaced with “X” to avoid problems in downstream analyses and treated as “NNN” at the nucleotide level. The amino acid sequences were aligned using MAFFT v.7.123b (Katoh and Standley, 2013) employing the *L-INS-i* algorithm. Subsequent alignment refinement and the removal of outlier sequences were conducted in accordance with the methodology outlined by Petersen *et al.* (2017), applied at both the amino acid and nucleotide levels. Reference species within the ortholog set were excluded, and corresponding nucleotide sequence alignments for all single-copy genes were generated using Pal2Nal (Suyama et al. 2006). Sections identified as randomly or ambiguously aligned by Aliscore v. 2.0 (Misof and Misof, 2009; Kück et al. 2010) at amino acid level—utilising parameters for the maximal number of pairwise comparisons and the option “-e” for handling gappy alignments derived from RNASeq data, alongside default settings—were removed using ALICUT v. 2.3 (<https://www.zfmk.de/en/research/research-centres-and-groups/utilities>). The corresponding regions in the nucleotide alignments were identified and removed respectively using a custom perl script. Leading and trailing gaps in each Multiple sequence alignment (MSA) were recoded as “X” for the amino acids and “N” for nucleotides and final concatenation was carried out with FASconCAT-G (Kück and Longo, 2014). To enhance the information content (IC) of our dataset, we applied MARE version 0.1.2-rc (Misof et al. 2013) to the amino acid supermatrix with the flag “-c” (using default parameters for other settings) to retain all species in the dataset.

**References**

Katoh K & Standley DM. 2013 “MAFFT multiple sequence alignment software version 7: improvements in performance and usability.” Molecular Biology and Evolution, 30(4), 772–780. DOI: 10.1093/molbev/mst010

Kück P & Longo GC .2014. "FASconCAT-G: extensive functions for multiple sequence alignment preparations concerning phylogenetic studies." Frontiers in Zoology, 18;11(1):81. DOI:10.1186/s12983-014-0081-x

Kück P, Meusemann K, Dambach J, Thormann B, von Reumont BM, Wägele JW & Misof B. 2010. "Parametric and non-parametric masking of randomness in sequence alignments can be improved and leads to better resolved trees." Frontiers in Zoology 7(1):10. DOI: 10.1186/1742-9994-7-10

Misof B, Meyer B, von Reumont BM, Kück P, Misof K & Meusemann K. 2013. "Selecting informative subsets of sparse supermatrices increases the chance to find correct trees." BMC bioinformatics, 14(1), 348.

Misof B & Misof K. 2009. "A Monte Carlo approach successfully identifies randomness in multiple sequence alignments: a more objective means of data exclusion." Systematic Biology, 58, 21–34.

Petersen M, Meusemann K, Donath A, Dowling D, Liu S, Peters RS, Podsiadlowski L, Vasilikopoulos A, Zhou X, Misof B & Niehuis O. 2017. "Orthograph: a versatile tool for mapping coding nucleotide sequences to clusters of orthologous genes." BMC Bioinformatics, 18, 111. DOI: 10.1186/s12859-017-1529-8

Suyama M, Torrents D, Bork P. 2006. “PAL2NAL: Robust conversion of protein sequence alignments into the corresponding codon alignments.” Nucleic Acids Research, 34(suppl_2): W609–12.
