# Supplementary material for: Phylogenomics resolves long-standing questions about the affinities of an endangered Corsican endemic fly
Source: J Insect Sci. 2024 Jul 25;24(4):9. doi: 10.1093/jisesa/ieae073 (PMC11271022; doi:10.1093/jisesa/ieae073)
Supplement: ieae073_suppl_Supplementary_Files_S2 [file ieae073_suppl_supplementary_files_s2.docx]

**Phylogenomics resolves long-standing questions about the affinities of an endangered Corsican endemic fly**

**Supplementary file 2. Morphological measurements of the specimens**

Dataset used to measure the body-size of *N. corsicana*, verifying it is the largest known rhinophorid. The dataset includes species name. specimen sex and location, body length and thoracic width (i.e., inter-tegular distance) measurements (mm).

***Materials and methods***

Measurements were performed using a Zeiss Axio Zoom V16 microscope equipped with a Axiocam 208 colour camera. Each measurement was repeated three times. The thoracic width and body length ratio were measured using the mean of the three measurements. Specifically, 14 specimens of *N. corsicana* were compared to 2 specimens of *Paykullia* *insularis* (Villeneuve)*,* 2 specimens *of P. nubilipennis* (Loew), 5 specimens of *P.* *partenopea* (Rondani), 14 specimens of *Phyto* *adolescens* Rondani, 2 specimens *of P. cingulata* (Zetterstedt)*,* 9 specimens *of P. melanocephala* (Meigen), 2 specimens of *Phyto* sp. [cf. *cingulata*], 3 specimens of *Rhinomorinia sarcophagina* (Schiner), 3 specimens of *Stevenia atramentaria* (Meigen), 5 specimens of *S. deceptoria* (Loew), 2 specimens of *S. etrusca* Cerretti & Pape*,* 7 specimens of *S. obscuripennis* (Loew), 3 specimens of *S. palermitana* Cerretti & Pape, 4 specimens of *S. signata* (Mik) and 3 specimens of *Tricogena rubricosa* (Meigen).

***Results***

The adult body size of *Nesodexia* is considerably larger, both in terms of body length and the thoracic width. Although specimens in the genus *Paykullia*, *Phyto* and *Stevenia*, especially of *S. etrusca* Cerretti & Pape, may occasionally reach a maximum length of 10.7 mm, none of them has an average width (measured between the tegulae) of 2.98 mm and a ratio between the width (measured between the tegulae) and body length of 0.33 mm. Specimens of *Phyto* sp. [cf. *cingulata*] also show a high thoracic width and body length ratio (0.31). This is due to the fact that the specimens analysed have a considerable thoracic width value compared to the body length. However, for these specimens the average body length of the specimens is 5.95 mm and that of the thoracic width is 1.85 mm. Thus, based on the available data, *N.corsicana* is to be considered the largest known rhinophorid.

| Species name | Specimen sex | Specimen location | 1° Body length (mm) | 2° Body length (mm) | 3° Body length (mm) | Mean Body length (mm) | 1° Thoracic width (mm) | 2° Thoracic width (mm) | 3° Thoracic width (mm) | Mean Thoracic width (mm) | Thoracic width/Body length ratio |
| --- | --- | --- | --- | --- | --- | --- | --- | --- | --- | --- | --- |
| *Nesodexia corsicana* (Villeneuve) | Male | Cognocoli-Monticchi. Southern Corsica. France 41° 51' 04.4'' N 8° 54' 00.8'' E 730m 4.VII.2021 P. Cerretti leg. | 7.709 | 7.704 | 7.791 | 7.7 | 2.446 | 2.451 | 2.439 | 2.5 | 0.33 |
| *Nesodexia corsicana* (Villeneuve) | Male | Cognocoli-Monticchi. Southern Corsica. France 41° 51' 04.4'' N 8° 54' 00.8'' E 730m 4.VII.2021 P. Cerretti leg. | 7.514 | 7.479 | 7.572 | 7.5 | 2.382 | 2.377 | 2.389 | 2.4 | 0.33 |

| *Nesodexia corsicana* (Villeneuve) | Male | Cognocoli-Monticchi. Southern Corsica. France 41° 51' 04.4'' N 8° 54' 00.8'' E 730m 4.VII.2021 P. Cerretti leg. | 9.509 | 9.525 | 9.482 | 9.5 | 2.995 | 2.953 | 3.012 | 3.0 | 0.33 |
| --- | --- | --- | --- | --- | --- | --- | --- | --- | --- | --- | --- |
| *Nesodexia corsicana* (Villeneuve) | Male | Cognocoli-Monticchi. Southern Corsica. France 41° 51' 04.4'' N 8° 54' 00.8'' E 730m 4.VII.2021 P. Cerretti leg. | 8.367 | 8.372 | 8.381 | 8.4 | 2.567 | 2.567 | 2.526 | 2.6 | 0.33 |
| *Nesodexia corsicana* (Villeneuve) | Male | Sisco. Upper Corsica. France 42° 48' N 09° 26' E 25-300m. 2.VII.2021 P. Cerretti leg. | 9.916 | 9.957 | 9.967 | 10.0 | 3.339 | 3.466 | 3.482 | 3.4 | 0.33 |
| *Nesodexia corsicana* (Villeneuve) | Male | Cognocoli-Monticchi. Southern Corsica. France 41° 51' 04.4'' N 8° 54' 00.8'' E 730m 4.VII.2021 P. Cerretti leg. | 8.812 | 8.852 | 8.855 | 8.8 | 2.83 | 2.863 | 2.788 | 2.8 | 0.33 |
| *Nesodexia corsicana* (Villeneuve) | Male | Cognocoli-Monticchi. Southern Corsica. France 41° 51' 04.4'' N 8° 54' 00.8'' E 730m 4.VII.2021 P. Cerretti leg. | 9.616 | 9.642 | 9.655 | 9.6 | 3.128 | 3.141 | 3.212 | 3.2 | 0.33 |

| *Nesodexia corsicana* (Villeneuve) | Male | Cognocoli-Monticchi. Southern Corsica. France 41° 51' 04.4'' N 8° 54' 00.8'' E 730m 4.VII.2021 P. Cerretti leg. | 8.759 | 8.635 | 8.759 | 8.7 | 2.979 | 2.937 | 2.945 | 3 | 0.33 |
| --- | --- | --- | --- | --- | --- | --- | --- | --- | --- | --- | --- |
| *Nesodexia corsicana* (Villeneuve) | Male | Cognocoli-Monticchi. Southern Corsica. France 41° 51' 04.4'' N 8° 54' 00.8'' E 730m 4.VII.2021 P. Cerretti leg. | 9.149 | 9.309 | 9.123 | 9.2 | 3.063 | 3.038 | 3.047 | 3.1 | 0.33 |
| *Nesodexia corsicana* (Villeneuve) | Male | Cognocoli-Monticchi. Southern Corsica. France 41° 51' 04.4'' N 8° 54' 00.8'' E 730m 4.VII.2021 P. Cerretti leg. | 9.529 | 9.36 | 9.671 | 9.5 | 3.096 | 3.122 | 3.164 | 3.1 | 0.33 |
| *Nesodexia corsicana* (Villeneuve) | Male | Cognocoli-Monticchi. Southern Corsica. France 41° 51' 04.4'' N 8° 54' 00.8'' E 730m 4.VII.2021 P. Cerretti leg. | 9.986 | 10.036 | 10.019 | 10 | 3.734 | 3.675 | 3.692 | 3.7 | 0.33 |
| *Nesodexia corsicana* (Villeneuve) | Male | Cognocoli-Monticchi. Southern Corsica. France 41° 51' 04.4'' N 8° 54' 00.8'' E 730m 4.VII.2021 P. Cerretti leg. | 9.108 | 9.117 | 9.128 | 9.1 | 2.811 | 2.82 | 2.878 | 2.8 | 0.33 |
| *Nesodexia corsicana* (Villeneuve) | Male | Cognocoli-Monticchi. Southern Corsica. France 41° 51' 04.4'' N 8° 54' 00.8'' E 730m 7.VII.2021 P. Cerretti leg. | 8.673 | 8.608 | 8.78 | 8.7 | 2.778 | 2.828 | 2.828 | 2.8 | 0.33 |
| *Nesodexia corsicana* (Villeneuve) | Male | Cognocoli-Monticchi. Southern Corsica. France 41° 51' 04.4'' N 8° 54' 00.8'' E 730m 4.VII.2021 P. Cerretti leg. | 10.054 | 10.034 | 10.073 | 10.1 | 3.367 | 3.281 | 3.281 | 3.3 | 0.33 |
| *Paykullia insularis* (Villeneuve) | Male | Cognocoli-Monticchi. Southern Corsica. France 41° 51' 04.4'' N 8° 54' 00.8'' E 730m 4.VII.2021 P. Cerretti leg. | 6.371 | 6.403 | 6.334 | 6.4 | 1.86 | 1.842 | 1.854 | 1.9 | 0.29 |
| *Paykullia insularis* (Villeneuve) | Male | Cognocoli-Monticchi. Southern Corsica. France 41° 51' 04.4'' N 8° 54' 00.8'' E 730m 4.VII.2021 P. Cerretti leg. | 6.422 | 6.309 | 6.393 | 6.4 | 1.856 | 1.8 | 1.825 | 1.8 | 0.29 |
| *Paykullia nubilipennis* (Loew) | Female | Licenza. Rome. Latium. Italy | 9.478 | 9.502 | 9.454 | 9.5 | 2.532 | 2.616 | 2.545 | 2.6 | 0.27 |

| *Paykullia nubilipennis (Loew)* | Female | Palermo. Sicily. Italy | 9.68 | 9.745 | 9.654 | 9.7 | 2.667 | 2.641 | 2.583 | 2.6 | 0.27 |
| --- | --- | --- | --- | --- | --- | --- | --- | --- | --- | --- | --- |
| *Paykullia partenopea (Rondani)* | Male | Palermo. Sicily. Italy | 6.075 | 6.123 | 6.086 | 6.1 | 1.665 | 1.626 | 1.652 | 1.7 | 0.27 |
| *Paykullia partenopea (Rondani)* | Male | L'Aquila. Abruzzo. Italy | 6.863 | 6.923 | 6.799 | 6.9 | 1.986 | 1.96 | 1.998 | 2 | 0.27 |
| *Paykullia partenopea (Rondani)* | Male | Rome. Latium. Italy | 6.038 | 6.034 | 6.017 | 6 | 1.6 | 1.626 | 1.613 | 1.6 | 0.27 |
| *Paykullia partenopea (Rondani)* | Female | Giglio Island. Tuscany. Italy | 5.92 | 5.897 | 5.906 | 5.9 | 1.401 | 1.414 | 1.407 | 1.4 | 0.27 |
| *Paykullia partenopea (Rondani)* | Male | Mammola Reggio Calabria 800m 11.V.2004 Hand net P. Cerretti D. Birtele G. Nardi D. Whitmore leg | 7.675 | 7.786 | 7.658 | 7.7 | 2.037 | 2.076 | 2.018 | 2 | 0.27 |
| *Phyto adolescens (Rondani)* | Male | Stagno di Marceddi. Oristano. Sardinia. Italy 25.V.2006 Hand net D. Whitmore M. Bardiani. D. Birtele P. Cornacchia leg. | 6.754 | 6.823 | 6.785 | 6.8 | 1.859 | 1.832 | 1.886 | 1.9 | 0.29 |

| *Phyto adolescens (Rondani)* | Male | Piano Zucchi. Madonie. Palermo. Sicily. Italy 37 52783 N 14 00147 E 1238m 21.V.2004 Hand net P. Cerretti D. Birtele G. Nardi D. Whitmore leg. | 6.756 | 6.836 | 6.813 | 6.8 | 2 | 2.034 | 2 | 2 | 0.29 |
| --- | --- | --- | --- | --- | --- | --- | --- | --- | --- | --- | --- |
| *Phyto adolescens (Rondani)* | Male | Bosco della Ficuzza. Torretta Torre. Palermo. Sicily. Italy 600-1000m 18.V.2004 Hand net P. Cerretti D. Birtele G. Nardi D. Whitmore leg. | 9.164 | 9.203 | 9.187 | 9.2 | 2.674 | 2.6 | 2.647 | 2.6 | 0.29 |
| *Phyto adolescens (Rondani)* | Female | Bosco della Ficuzza. Rocca Busambra south side. Palermo. Sicily. Italy 37.50826 N 13.21572 E 716m 7.V.2004 Hand net P. Cerretti D. Birtele G. Nardi D. Whitmore leg. | 8.2 | 8.142 | 8.236 | 8.2 | 2.277 | 2.364 | 2.364 | 2.3 | 0.29 |
| *Phyto adolescens (Rondani)* | Male | Bosco della Ficuzza. Rocca Busambra south side. Palermo. Sicily. Italy 37.50826 N 13.21572 E 716m 7.V.2004 Hand net P. Cerretti D. Birtele G. Nardi D. Whitmore leg. | 7.203 | 7.294 | 7.156 | 7.2 | 2.068 | 2.108 | 2.095 | 2.1 | 0.29 |

| *Phyto adolescens (Rondani)* | Male | Oasi dello Zingaro. Trapani. Sicily. Italy 9.V.2004 P. Cerretti D. Birtele G. Nardi D. Whitmore leg. | 9.786 | 9.768 | 9.734 | 9.8 | 2.836 | 2.755 | 2.795 | 2.8 | 0.29 |
| --- | --- | --- | --- | --- | --- | --- | --- | --- | --- | --- | --- |
| *Phyto adolescens (Rondani)* | Male | Bosco della Ficuzza. Valle Cerasa. Palermo. Sicily. Italy UTM 33 S 362011 4190550 28.VI.2004 Hand net P. Cerretti leg. | 6.896 | 6.932 | 6.876 | 6.9 | 2.074 | 2.154 | 2.111 | 2.1 | 0.29 |
| *Phyto adolescens (Rondani)* | Male | Bosco della Ficuzza. Valle Cerasa. Palermo. Sicily. Italy UTM 33 S 362011 4190550 28.VI.2004 Hand net P. Cerretti leg. | 5.827 | 5.765 | 5.812 | 5.8 | 1.653 | 1.724 | 1.699 | 1.7 | 0.29 |
| *Phyto adolescens (Rondani)* | Male | Oasi dello Zingaro. Trapani. Sicily. Italy 9.V.2004 P. Cerretti D. Birtele G. Nardi D. Whitmore leg. | 7.475 | 7.576 | 7.387 | 7.5 | 2.192 | 2.263 | 2.224 | 2.2 | 0.29 |
| *Phyto adolescens (Rondani)* | Female | Bosco della Ficuzza. Torretta Torre. Palermo. Sicily. Italy 600-1000m 18.V.2004 Hand net P. Cerretti D. Birtele G. Nardi D. Whitmore leg. | 8.556 | 8.607 | 8.573 | 8.6 | 2.69 | 2.627 | 2.735 | 2.7 | 0.29 |

| *Phyto adolescens (Rondani)* | Male | Bosco della Ficuzza. Rocca Busambra south side. Palermo. Sicily. Italy 37.50826 N 13.21572 E 716m 7.V.2004 Hand net P. Cerretti D. Birtele G. Nardi D. Whitmore leg. | 7.302 | 7.421 | 7.287 | 7.3 | 2.114 | 2.095 | 2.12 | 2.1 | 0.29 |
| --- | --- | --- | --- | --- | --- | --- | --- | --- | --- | --- | --- |
| *Phyto adolescens (Rondani)* | Female | Piano Zucchi. Madonie. Palermo. Sicily. Italy 37 52783 N 14 00147 E 1238m 21.V.2004 Hand net P. Cerretti D. Birtele G. Nardi D. Whitmore leg. | 8.452 | 8.541 | 8.503 | 8.5 | 2.496 | 2.482 | 2.527 | 2.5 | 0.29 |
| *Phyto adolescens (Rondani)* | Female | Piano Zucchi. Madonie. Palermo. Sicily. Italy 37 52783 N 14 00147 E 1238m 21.V.2004 Hand net P. Cerretti D. Birtele G. Nardi D. Whitmore leg. | 8.206 | 8.198 | 8.243 | 8.2 | 2.494 | 2.487 | 2.538 | 2.5 | 0.29 |
| *Phyto adolescens (Rondani)* | Female | Bosco della Ficuzza. Valle Cerasa. Palermo. Sicily. Italy UTM 33 S 362011 4190550 28.VI.2004 Hand net P. Cerretti leg. | 6.811 | 6.789 | 6.834 | 6.8 | 1.905 | 1.892 | 1.848 | 1.9 | 0.29 |

| *Phyto cingulata (Zetterstedt)* | Female | Iglesias. Marganai Mountains Tintillonis. Sardinia. Italy 39.20452 N 8.33746 E 480m 09.VI.2004 Hand net P. Cerretti D. Birtele G. Nardi D. Whitmore leg. | 5.17 | 5.231 | 5.187 | 5.2 | 1.507 | 1.541 | 1.562 | 1.5 | 0.28 |
| --- | --- | --- | --- | --- | --- | --- | --- | --- | --- | --- | --- |
| *Phyto cingulata (Zetterstedt)* | Female | Arbus. Marina di Arbus. Medio Campidano. Sardinia. Italy 25.V.2006 Hand net D. Whitmore M. Bardiani. D. Birtele P. Cornacchia leg. | 6.459 | 6.513 | 6.487 | 6.5 | 1.847 | 1.799 | 1.792 | 1.8 | 0.28 |
| *Phyto melanocephala (Meigen)* | Female | Tor Sapienza. Rome. Latium. Italy 6.IV.2019 M. Mei leg. | 8.852 | 8.972 | 8.831 | 8.9 | 2.53 | 2.588 | 2.611 | 2.6 | 0.29 |
| *Phyto melanocephala (Meigen)* | Female | Tor Sapienza. Rome. Latium. Italy 6.IV.2019 M. Mei leg. | 8.253 | 8.302 | 8.345 | 8.3 | 2.528 | 2.52 | 2.528 | 2.5 | 0.29 |
| *Phyto melanocephala (Meigen)* | Female | Piano Zucchi. Madonie. Palermo. Sicily. Italy 37 52783 N 14 00147 E 1238m 21.V.2004 Hand net P. Cerretti D. Birtele G. Nardi D. Whitmore leg. | 7.395 | 7.403 | 7.441 | 7.4 | 2.201 | 2.261 | 2.261 | 2.2 | 0.29 |
| *Phyto melanocephala (Meigen)* | Male | Tor Sapienza. Rome. Latium. Italy 25.V.2019 M. Mei leg. | 8.439 | 8.387 | 8.405 | 8.4 | 2.316 | 2.288 | 2.248 | 2.3 | 0.29 |
| *Phyto melanocephala (Meigen)* | Female | Tor Sapienza. Rome. Latium. Italy 25.V.2019 M. Mei leg. | 7.804 | 7.765 | 7.841 | 7.8 | 2.376 | 2.363 | 2.329 | 2.4 | 0.29 |
| *Phyto melanocephala (Meigen)* | Female | Tor Sapienza. Rome. Latium. Italy 25.V.2019 M. Mei leg. | 7.227 | 7.132 | 7.254 | 7.2 | 1.942 | 1.942 | 1.969 | 2 | 0.29 |
| *Phyto melanocephala (Meigen)* | Female | Tor Sapienza. Rome. Latium. Italy 25.V.2019 M. Mei leg. | 6.68 | 6.732 | 6.696 | 6.7 | 1.983 | 2.003 | 1.956 | 2 | 0.29 |
| *Phyto melanocephala (Meigen)* | Female | Tor Sapienza. Rome. Latium. Italy 25.V.2019 M. Mei leg. | 5.713 | 5.658 | 5.823 | 5.7 | 1.643 | 1.609 | 1.596 | 1.6 | 0.29 |
| *Phyto melanocephala (Meigen)* | Male | Tor Sapienza. Rome. Latium. Italy 25.V.2019 M. Mei leg. | 5.608 | 5.69 | 5.546 | 5.6 | 1.555 | 1.637 | 1.589 | 1.6 | 0.29 |
| *Phyto sp. [cf. cingulata]* | Male | Toccone. Upper Corsica. France 42° 34' 59.3'' N 9° 03' 24.6'' E 620m 6.VII.2021 P. Cerretti leg. | 6.564 | 6.587 | 6.623 | 6.6 | 1.923 | 1.944 | 1.915 | 1.9 | 0.31 |
| *Phyto sp. [cf. cingulata]* | Female | Cognocoli-Monticchi. Southern Corsica. France 41° 51' 04.4'' N 8° 54' 00.8'' E 730m 4.VII.2021 P. Cerretti leg. | 5.303 | 5.298 | 5.243 | 5.3 | 1.78 | 1.745 | 1.773 | 1.8 | 0.31 |

| *Rhinomorinia sarcophagina (Schiner)* | Male | Mammola Reggio Calabria 800m 11.V.2004 Hand net P. Cerretti D. Birtele G. Nardi D. Whitmore leg. | 6.995 | 7.132 | 6.876 | 7 | 2.075 | 1.974 | 2.063 | 2 | 0.3 |
| --- | --- | --- | --- | --- | --- | --- | --- | --- | --- | --- | --- |
| *Rhinomorinia sarcophagina (Schiner)* | Male | Mammola Reggio Calabria 800m 11.V.2004 Hand net P. Cerretti D. Birtele G. Nardi D. Whitmore leg. | 6.768 | 6.798 | 6.732 | 6.8 | 2.15 | 2.061 | 2.19 | 2.1 | 0.3 |
| *Rhinomorinia sarcophagina (Schiner)* | Male | Mammola Reggio Calabria 800m 11.V.2004 Hand net P. Cerretti D. Birtele G. Nardi D. Whitmore leg. | 6.809 | 6.735 | 6.944 | 6.8 | 1.974 | 2.015 | 1.974 | 2 | 0.3 |
| *Stevenia atramentaria (Meigen)* | Male | Peloponneso Elide - Ilia prov. Neohori. Greece 37 51792 N 21 61389 E 40m 20.IV.2003 Hand net P. Cerretti L. Facchinelli M. Tisato L. Valerio S. Vanin leg. | 7.596 | 7.613 | 7.701 | 7.6 | 1.936 | 1.951 | 1.988 | 2 | 0.27 |
| *Stevenia atramentaria (Meigen)* | Male | Sterea Ellada Fthiotida prov. Thermopyles 38 79292 N 22 53294 E 70m 27.IV.2003 Hand net P. Cerretti L. Facchinelli M. Tisato L. Valerio S. Vanin leg. | 6.971 | 7.125 | 6.956 | 7 | 1.943 | 1.949 | 1.91 | 1.9 | 0.27 |
| *Stevenia atramentaria (Meigen)* | Male | Sterea Ellada Fthiotida prov. Thermopyles 38 79292 N 22 53294 E 70m 27.IV.2003 Hand net P. Cerretti L. Facchinelli M. Tisato L. Valerio S. Vanin leg. | 6.343 | 6.318 | 6.287 | 6.3 | 1.658 | 1.684 | 1.723 | 1.7 | 0.27 |
| *Stevenia deceptoria (Loew)* | Female | Carbonia- Iglesias Conca Margiani Sardinia. Italy UTM WGS84 32S 0462470 4357011 725m 7.IX.2006 D.Avesani M. Bardiani. D. Birtele G. Nardi leg. | 7.674 | 7.713 | 7.734 | 7.7 | 1.904 | 1.91 | 1.93 | 1.9 | 0.24 |
| *Stevenia deceptoria (Loew)* | Female | Carbonia Iglesias Domusnovas Valle Oridda. Sardinia. Italy UTM WGS84 32 S 0466973 4362228 592m 15.VII.2006 D.Avesani M. Bardiani. D. Birtele G. Nardi P. Cerretti D. Whitmore M. Mei leg. | 7.67 | 7.821 | 7.657 | 7.7 | 1.981 | 1.91 | 1.911 | 1.9 | 0.24 |
| *Stevenia deceptoria (Loew)* | Male | Piano Zucchi. Madonie. Palermo. Sicily 1238m N 37 52783 E 14 00147 21.V.2004 Hand net P. Cerretti D. Birtele G. Nardi D. Whitmore leg. | 8.545 | 8.615 | 8.687 | 8.6 | 1.938 | 1.859 | 1.858 | 1.9 | 0.24 |
| *Stevenia deceptoria (Loew)* | Female | Piano Zucchi. Madonie. Palermo. Sicily 1238m N 37 52783 E 14 00147 21.V.2004 Hand net P. Cerretti D. Birtele G. Nardi D. Whitmore leg. | 9.025 | 9.104 | 8.973 | 9 | 2.345 | 2.353 | 2.416 | 2.4 | 0.24 |
| *Stevenia deceptoria (Loew)* | Female | Piano Zucchi. Madonie. Palermo. Sicily 1238m N 37 52783 E 14 00147 21.V.2004 Hand net P. Cerretti D. Birtele G. Nardi D. Whitmore leg. | 6.264 | 6.321 | 6.342 | 6.3 | 1.524 | 1.486 | 1.454 | 1.5 | 0.24 |
| *Stevenia etrusca Cerretti & Pape* | Female | Scarlino Cala di Terra Rossa Grosseto. Tuscany 02.VI.2006 Hand net P. Cerretti D. Birtele G. Nardi M. Tosato D. Whitmore leg. | 10.247 | 10.302 | 10.296 | 10.3 | 2.416 | 2.44 | 2.434 | 2.4 | 0.25 |
| *Stevenia etrusca Cerretti & Pape* | Female | Scarlino Cala di Terra Rossa Grosseto. Tuscany 02.VI.2006 Hand net P. Cerretti D. Birtele G. Nardi M. Tosato D. Whitmore leg. | 10.668 | 10.723 | 10.698 | 10.7 | 2.757 | 2.764 | 2.751 | 2.8 | 0.25 |

| *Stevenia obscuripennis (Loew)* | Female | Gioiosa Ionica. Reggio Calabria. Italy 38 20913 N 16 16815 E(on *Thapsia garganica*) 12.V.2004 Hand net P. Cerretti D. Birtele G. Nardi D. Whitmore leg. | 6.818 | 6.768 | 6.856 | 6.8 | 1.648 | 1.648 | 1.677 | 1.7 | 0.26 |
| --- | --- | --- | --- | --- | --- | --- | --- | --- | --- | --- | --- |
| *Stevenia obscuripennis (Loew)* | Female | Tor Sapienza. Rome. Latium. Italy 6.IV.2019 M. Mei leg. | 7.126 | 7.098 | 7.156 | 7.1 | 1.878 | 1.923 | 1.872 | 1.9 | 0.26 |
| *Stevenia obscuripennis (Loew)* | Female | Tor Sapienza. Rome. Latium. Italy 6.IV.2019 M. Mei leg. | 7.041 | 7.113 | 6.976 | 7 | 1.697 | 1.684 | 1.684 | 1.7 | 0.26 |
| *Stevenia obscuripennis (Loew)* | Female | Tor Sapienza. Rome. Latium. Italy 6.IV.2019 M. Mei leg. | 6.205 | 6.312 | 6.202 | 6.2 | 1.872 | 1.807 | 1.801 | 1.8 | 0.26 |
| *Stevenia obscuripennis (Loew)* | Female | Tor Sapienza. Rome. Latium. Italy 6.IV.2019 M. Mei leg. | 7.525 | 7.487 | 7.564 | 7.5 | 1.807 | 1.8 | 1.736 | 1.8 | 0.26 |
| *Stevenia obscuripennis (Loew)* | Female | Tor Sapienza. Rome. Latium. Italy 6.IV.2019 M. Mei leg. | 7.193 | 7.234 | 7.208 | 7.2 | 1.769 | 1.82 | 1.761 | 1.8 | 0.26 |
| *Stevenia obscuripennis (Loew)* | Female | Tor Sapienza. Rome. Latium. Italy 6.IV.2019 M. Mei leg. | 6.272 | 6.342 | 6.306 | 6.3 | 1.593 | 1.619 | 1.567 | 1.6 | 0.26 |

| *Stevenia palermitana Cerretti & Pape* | Male | Bosco della Ficuzza. Torretta Torre. Palermo. Sicily. Italy 600-1000m 18.V.2004 Hand net P. Cerretti D. Birtele G. Nardi D. Whitmore leg. | 6.902 | 7.005 | 6.903 | 6.9 | 1.793 | 1.732 | 1.75 | 1.8 | 0.27 |
| --- | --- | --- | --- | --- | --- | --- | --- | --- | --- | --- | --- |
| *Stevenia palermitana Cerretti & Pape* | Male | Bosco della Ficuzza. Torretta Torre. Palermo. Sicily. Italy 600-1000m 18.V.2004 Hand net P. Cerretti D. Birtele G. Nardi D. Whitmore leg. | 7.309 | 7.277 | 7.321 | 7.3 | 1.935 | 1.942 | 1.991 | 2 | 0.27 |
| *Stevenia palermitana Cerretti & Pape* | Female | Bosco della Ficuzza. Torretta Torre. Palermo. Sicily. Italy 600-1000m 18.V.2004 Hand net P. Cerretti D. Birtele G. Nardi D. Whitmore leg. | 6.791 | 6.886 | 6.765 | 6.8 | 1.851 | 1.874 | 1.751 | 1.8 | 0.27 |
| *Stevenia signata (Mik)* | Female | Peloponneso Elide - Ilia prov. Neohori. Greece 37 51792 N 21 61389 E 40m 20.IV.2003 Hand net P. Cerretti L. Facchinelli M. Tisato L. Valerio S. Vanin leg. | 8.461 | 8.542 | 8.523 | 8.5 | 1.961 | 2.024 | 1.981 | 2 | 0.24 |

| *Stevenia signata (Mik)* | Female | Attica - Attiki prov. Anavyssos. Greece 37 77484 N 23 93184 E 260m 26.IV.2003 Hand net P. Cerretti L. Facchinelli M. Tisato L. Valerio S. Vanin leg. | 7.891 | 7.964 | 7.912 | 7.9 | 1.946 | 1.996 | 1.981 | 2 | 0.24 |
| --- | --- | --- | --- | --- | --- | --- | --- | --- | --- | --- | --- |
| *Stevenia signata (Mik)* | Female | Sterea Ellada Viotia prov. Near Thiva Greece 38 41005 N 23 21922 E 140m 26.IV.2003 Hand net P. Cerretti L. Facchinelli M. Tisato L. Valerio S. Vanin leg. | 8.232 | 8.211 | 8.245 | 8.2 | 1.882 | 1.804 | 1.854 | 1.9 | 0.24 |
| *Stevenia signata (Mik)* | Female | Attica - Attiki prov. Anavyssos. Greece 37 77484 N 23 93184 E 260m 26.IV.2003 Hand net P. Cerretti L. Facchinelli M. Tisato L. Valerio S. Vanin leg. | 7.145 | 7.213 | 7.232 | 7.2 | 1.627 | 1.662 | 1.648 | 1.7 | 0.24 |
| *Tricogena rubricosa (Meigen)* | Female | 30 Km SE Tunis. Tunisia 30.IV.1988 | 5.595 | 5.643 | 5.622 | 5.6 | 1.558 | 1.569 | 1.528 | 1.6 | 0.27 |
| *Tricogena rubricosa (Meigen)* | Female | 30 Km SE Tunis. Tunisia 30.IV.1988 | 7.566 | 7.643 | 7.612 | 7.6 | 1.915 | 1.927 | 1.933 | 1.9 | 0.27 |

| *Tricogena rubricosa (Meigen)* | Female | Åbrolla. Osby. Sweden 56° 26' N 14° 08' E 142m 21.IX.2014 V. Michelsen | 6.008 | 5.963 | 6.053 | 6 | 1.622 | 1.605 | 1.605 | 1.6 | 0.27 |
| --- | --- | --- | --- | --- | --- | --- | --- | --- | --- | --- | --- |

**Thoracic width and body length ratio per species. *Nesodexia corsicana* shows the greatest value when compared with other rhinophorine species.**
