# Supplementary material for: Phylogenomics resolves long-standing questions about the affinities of an endangered Corsican endemic fly
Source: J Insect Sci. 2024 Jul 25;24(4):9. doi: 10.1093/jisesa/ieae073 (PMC11271022; doi:10.1093/jisesa/ieae073)
Supplement: ieae073_suppl_Supplementary_Files_S3 [file ieae073_suppl_supplementary_files_s3.docx]

**Phylogenomics resolves long-standing questions about the affinities of an endangered Corsican endemic fly**

**Supplementary file 3. Conservation assessment**

***Hypergeometric distribution test***

The AOH map was evaluated within the EOO of *N. corsicana*. Species-habitat links to the CORINE land-cover categories are based on knowledge of species’ habitat requirements as observed from the occurrence records. We evaluated the map as better than expected under randomness using a hypergeometric distribution approach (Jiménez and Soberón, 2020). The hypergeometric distribution allows testing for accuracy of species distribution models using presence-only data. Given a defined number of occurrences, the test describes the probability of each occurrence point to fall within the mapped habitat of a species. We defined a confidence interval of 95%, calculated point prevalence and model prevalence and consequently the upper and lower limits of the hypergeometric function. The model performance is evaluated through the calculation of the point prevalence in relation to the upper limit of the function. The model can be considered better than random if the model prevalence exceeds the upper limit of the function. The point prevalence is the proportion of occurrence points out of the total number of occurrences within the range of the species falling inside the available habitat. For the map validation, we used all occurrence points of *N. corsicana*. Before running the hypergeometric distribution test, we applied a buffer of 150 m to the species’ occurrence points. This was done to avoid errors due to possible inaccuracy of the occurrence records and errors due to the resolution of the land cover map map (100 m). We report a summary of the hypergeometric distribution test:

*Nesodexia corsicana*

confidence interval 0.95

n° of points 7

upper and lower limit 6 ; 1

n° of points inside the model 6

upper limit 85.71 %

point prevalence 85.71 %

model prevalence 47.74 %

***Species-habitat land cover scores***

The following table shows the CORINE land cover categories and the correspondent assigned score. Categories with an assigned score of 1 represent habitat for the species. Categories with an assigned score of 0 do not represent habitat for the species.

| **Land cover code** | **Land-cover category** | **Habitat score** |
| --- | --- | --- |
| 1 | 111 - Continuous urban fabric | 0 |
| 2 | 112 - Discontinuous urban fabric | 0 |
| 3 | 121 - Industrial or commercial units | 0 |
| 4 | 122 - Road and rail networks and associated land | 0 |
| 5 | 123 - Port areas | 0 |
| 6 | 124 - Airports | 0 |
| 7 | 131 - Mineral extraction sites | 0 |
| 8 | 132 - Dump sites | 0 |
| 9 | 133 - Construction sites | 0 |
| 10 | 141 - Green urban areas | 0 |
| 11 | 142 - Sport and leisure facilities | 0 |
| 12 | 211 - Non-irrigated arable land | 0 |
| 13 | 212 - Permanently irrigated land | 0 |
| 14 | 213 - Rice fields | 0 |
| 15 | 221 - Vineyards | 0 |
| 16 | 222 - Fruit trees and berry plantations | 0 |
| 17 | 223 - Olive groves | 0 |
| 18 | 231 - Pastures | 0 |
| 19 | 241 - Annual crops associated with permanent crops | 0 |
| 20 | 242 - Complex cultivation patterns | 0 |
| 21 | 243 - Land principally occupied by agriculture with significant areas of natural vegetation | 0 |
| 22 | 244 - Agro-forestry areas | 0 |
| 23 | 311 - Broad-leaved forest | 1 |
| 24 | 312 - Coniferous forest | 1 |
| 25 | 313 - Mixed forest | 1 |
| 26 | 321 - Natural grasslands | 0 |
| 27 | 322 - Moors and heathland | 0 |
| 28 | 323 - Sclerophyllous vegetation | 1 |
| 29 | 324 - Transitional woodland-shrub | 0 |
| 30 | 331 - Beaches - dunes - sands | 0 |
| 31 | 332 - Bare rocks | 0 |
| 32 | 333 - Sparsely vegetated areas | 0 |
| 33 | 334 - Burnt areas | 0 |
| 34 | 335 - Glaciers and perpetual snow | 0 |
| 35 | 411 - Inland marshes | 0 |
| 36 | 412 - Peat bogs | 0 |
| 37 | 421 - Salt marshes | 0 |
| 38 | 422 - Salines | 0 |
| 39 | 423 - Intertidal flats | 0 |
| 40 | 511 - Water courses | 0 |
| 41 | 512 - Water bodies | 0 |
| 42 | 521 - Coastal lagoons | 0 |
| 43 | 522 - Estuaries | 0 |
| 44 | 523 - Sea and ocean | 0 |
| 48 | 999 - NODATA | 0 |

**Species occurrence points**

| **Species** | **Latitude** | **Longitude** | **Elevation** | **Locality** | **Year** |
| --- | --- | --- | --- | --- | --- |
| *Nesodexia corsicana* | 42.8 | 9.43333 |  | Sisco | 2021 |
| *Nesodexia corsicana* | 41.85122 | 8.90002 | 730 | Cognocoli-Monticchi | 2021 |
| *Nesodexia corsicana* | 42.2619 | 8.8291 |  | Porto | 1967 |
| *Nesodexia corsicana* | 41.761 | 9.227 | 1244 | Zonza, Samulaghia | 2019 |
| *Nesodexia corsicana* | 41.76114 | 9.22581 | 1231 | Zonza, Samulaghia | 2019 |
| *Nesodexia corsicana* | 42.818467 | 9.434145 | 224 | Chioso | 2021 |
| *Nesodexia corsicana* | 41.914609 | 8.885791 |  | Campo di Loro | 1907 |

**Area of habitat map for *Nesodexia corsicana***
